# Computational approach for calculating the probability of eukaryotic translation initiation from ribo-seq data that takes into account leaky scanning

**DOI:** 10.1186/s12859-014-0380-4

**Published:** 2014-11-21

**Authors:** Audrey M Michel, Dmitry E Andreev, Pavel V Baranov

**Affiliations:** School of Biochemistry and Cell Biology, University College Cork, Cork, Ireland; Belozersky Institute of Physico-Chemical Biology, Lomonosov Moscow State University, Moscow, 119234 Russia

**Keywords:** Leaky scanning, Non-AUG initiation, Ribosome profiling, Translation initiation, 5′ leader, 5′ UTR TISs, Kozak context, uORF

## Abstract

**Background:**

Ribosome profiling (ribo-seq) provides experimental data on the density of elongating or initiating ribosomes at the whole transcriptome level that can be potentially used for estimating absolute levels of translation initiation at individual Translation Initiation Sites (TISs). These absolute levels depend on the mutual organisation of TISs within individual mRNAs. For example, according to the leaky scanning model of translation initiation in eukaryotes, a strong TIS downstream of another strong TIS is unlikely to be productive, since only a few scanning ribosomes would be able to reach the downstream TIS. In order to understand the dependence of translation initiation efficiency on the surrounding nucleotide context, it is important to estimate the strength of TISs independently of their mutual organisation, i.e. to estimate with what probability a ribosome would initiate at a particular TIS.

**Results:**

We designed a simple computational approach for estimating the probabilities of ribosomes initiating at individual start codons using ribosome profiling data. The method is based on the widely accepted leaky scanning model of translation initiation in eukaryotes which postulates that scanning ribosomes may skip a start codon if the initiation context is unfavourable and continue on scanning. We tested our approach on three independent ribo-seq datasets obtained in mammalian cultured cells.

**Conclusions:**

Our results suggested that the method successfully discriminates between weak and strong TISs and that the majority of numerous non-AUG TISs reported recently are very weak. Therefore the high frequency of non-AUG TISs observed in ribosome profiling experiments is due to their proximity to mRNA 5′-ends rather than their strength. Detectable translation initiation at non-AUG codons downstream of AUG codons is comparatively infrequent. The leaky scanning method will be useful for the characterization of differences in start codon selection between tissues, developmental stages and in response to stress conditions.

**Electronic supplementary material:**

The online version of this article (doi:10.1186/s12859-014-0380-4) contains supplementary material, which is available to authorized users.

## Background

Most of what we know about sequence dependent regulation of translation initiation in eukaryotes comes from careful experimental analyses that involve site-directed mutagenesis of nucleotide sequences derived from specific genes. The main drawback of such an approach is that it provides information that is context specific. It is often difficult to determine whether the observations relate to a particular gene or whether they can be extrapolated to all other genes as well. With the newly developed ribosome profiling technique (ribo-seq) [1], it is possible to look at the translation of all mRNAs in the cell simultaneously (see [2] for a review). Therefore the analysis of ribo-seq data can help us to discriminate general aspects of translational regulation from gene specific regulatory mechanisms.

**Figure 1:**
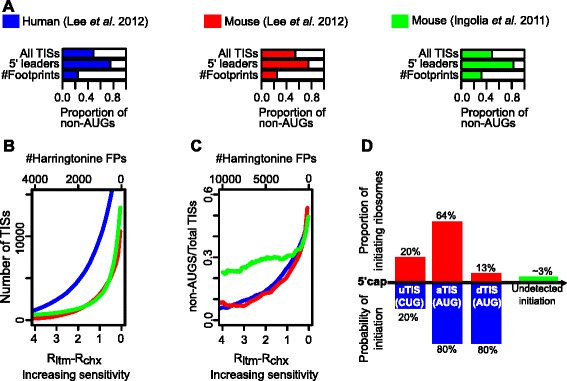
**The relative frequency of non-AUG TISs depends on the sensitivity of the methods and location relative to AUG TISs. A**. The frequencies of non-AUG codons among all TISs (top bars) and in 5′ leaders, i.e. upstream of annotated TISs, (middle bars) for the three ribo-seq datasets. The bottom bars “# Footprints” represent the proportion of footprint reads detected at all non-AUG TISs. **B**. The overall number of detected TISs correlates with the sensitivity (i.e. as the TIS detection threshold is decreased). **C**. The proportion of non-AUG TISs also correlates with the sensitivity. For A,B,C, the distributions were generated from initiating ribosome footprint data from Lee et al. [4] (blue Human, red Mouse) and Ingolia et al. [3] (green Mouse). **D**. A hypothetical example is given of an mRNA with one weak (20% efficient, first blue peak) and two strong (80% efficient, last two blue peaks) TISs. Because ribosomes encounter the weak upstream TIS first, the proportion of ribosomes initiating at the first TIS is higher than that for the third downstream (strong) TIS. The green bar represents all undetected TISs downstream of the most 3′ detectable TIS.

Three recently published ribosome profiling studies provided large datasets of initiating ribosomes footprints mapped to the transcriptomes of human and mouse cells [3-5]. Despite differences in the experimental approaches and computational techniques, the studies converged in the conclusion that approximately half of the TISs are non-AUGs (Figure 1A “All TISs”). This was unanticipated as the translation initiation machinery was thought to stringently select AUG codons for initiation, whereas non-AUG TISs were thought to be rare and gene-specific. While the ability of ribosomes to initiate at non-AUG codons was documented a long time ago [6-8] and functional non-AUG initiators have been identified using phylogenetic analysis [9], the efficiency of non-AUG initiation in general was expected to be very low. Earlier measurements of translation initiation in mammalian cell free extracts indicated that, even in optimal nucleotide context, non-AUG initiation efficiency is at least 20 times lower than that of AUG [10] and even lower in yeast [11].

Interestingly, the majority of TISs upstream of annotated TISs, i.e. within mRNA 5′ leaders (historically termed Untranslated Terminal Regions or UTRs), are non-AUGs (Figure 1A “5′ leaders”): ~83% in Ingolia et al. [3]; ~74% in Lee et al. [4]; 78% in Fritsch et al. [5]. This observation, however, is consistent with the widely accepted leaky scanning model of translation initiation in eukaryotes (discussed in [12]). In the case of the leaky scanning model, the preinitiation complex first assembles at the 5′end of the mRNA, and then scans the mRNA in the 5′ to the 3′ direction. Once the preinitiation complex encounters an initiation codon in a suitable context, a chain of events triggers the 60S subunit joining and the start of elongation. However, in suboptimal contexts, only a proportion of the ribosomes engage in initiating protein synthesis while the rest of the ribosomes continue scanning. As suggested by Lee et al. [4], in this scenario it is expected that low efficient TISs would be detected mostly upstream of strong TISs and not downstream. Therefore, in order to study the sequence properties determining the strength of a TIS, it is important to estimate its strength independent of its location relative to other TISs. For this purpose we designed a method for calculating translation initiation probabilities from the absolute values of translation initiation. We tested our approach on three publicly available datasets and have shown that the estimated probabilities of initiation at non-AUG TISs are typically much lower than those at AUG TISs. In addition to resolving the controversy, our approach can be useful for characterizing future ribo-seq datasets.

## Results

### How to measure the strength of a translation initiation site (TIS)?

One way to estimate the strength of a TIS is by counting the ribosomes that initiate at this particular codon. This is the basis of the signal generated in the ribo-seq studies described in the Background section [3-5]: the number of RNA fragments protected by the ribosomes arrested at a TIS should reflect the number of ribosomes initiating at this site. As the sensitivity of the experimental technique increases, the number of detected TISs should obviously increase and this indeed can be seen in Figure 1B. However, the number of ribosomes at a particular TIS largely depends on other factors such as mRNA levels and the rate at which the preinitiation complex is formed at the 5′-cap. Consequently, and counterintuitively, the number of ribosomes initiating at a TIS is not a good measure of TIS strength.

Figure 1C illustrates how the proportion of non-AUG TISs increases with increasing sensitivity. Hence, assigning a codon as an initiation site is also dependent on the ribosome footprint coverage thresholds that are used to distinguish starts from non-starts.

An alternative method that we explored in this work was to measure the strength of a TIS as the probability with which a ribosome initiates at the start.

### Proportion of the Absolute Signal (PAS) method

How to calculate the probability with which a ribosome would initiate at a particular TIS? Without any prior knowledge regarding the mechanism of translation initiation, one may suggest to define it as the relative frequency at which ribosomes initiate. Instead of asking the question regarding the number of detectable TISs, let us ask the question what is the chance that a ribosome would initiate at a non-AUG codon? To answer this question we simply need to count the number of ribosome protected fragments that have AUG and non-AUG codons in the position of the inferred peptidyl tRNA site (P-site). Only about a quarter or less of the ribosome footprints originate from non-AUG starts (Figure 1A “# Footprints”). The difference between “All TISs” and “# Footprints” distributions in Figure 1A is because TISs are not all equally efficient translation initiators. Purely in terms of the number of ribosome footprints, AUG codons appear to be more efficient in capturing initiating ribosomes than non-AUG codons which is in agreement with previous observations that AUG codons are better initiators. However, because of differences in the levels of mRNA, this comparison of AUG and non-AUG initiation is likely to be very inaccurate.

Because transcript levels and the rates of ribosomal load are different for each gene, the comparison of relative initiation strength is meaningful only within the same gene. The strength of a TIS can then be measured as the fraction of footprints that initiate at the TIS from the total number of footprints that initiate in the entire mRNA. So for an mRNA with initiating ribosome footprint reads (R_i_) at initiation sites TIS_1_, TIS_2_, TIS_3_ … TIS_k_, the probability of initiating footprints (P_i_) at each TIS_i_ can be calculated as follows:

1 $$ {P}_i=\frac{R_i}{{\displaystyle \sum_{s=1}^k{R}_s}} $$

Using the PAS approach (equation (1)), however, is inaccurate because it does not reflect the process of initiation codon selection by the scanning ribosome. This is illustrated with a hypothetical example given in Figure 1D which shows an mRNA with one weak (20% efficient, first blue peak) and two strong (80% efficient, last two blue peaks) TISs. Because ribosomes encounter the weak TIS first, the proportion of ribosomes initiating at the weak TIS (first red peak) is higher than that for the third (strong) TIS (third red peak). In other words, the number of ribosomes that are available to initiate at the third TIS is dependent on the efficiencies of the upstream TISs. Therefore, the proportion of ribosome footprints that are detected for the third TIS using the PAS approach (equation (1)) is not reflective of the TIS true strength. For the same reason, the frequency of non-AUG TISs is much higher within the 5′ leaders of mRNAs (Figure 1A “5′ leaders”).

### The Leaky Scanning (LS) method

To measure the strength of a TIS irrespective of its position within an mRNA, we devised a method that is based on the leaky scanning (LS) model of translation initiation. Consider an mRNA with initiation sites TIS_1_, TIS_2_, TIS_3_ … TIS_k_. Taking into account the leaky scanning mechanism, the number of footprint reads (R_i_) detected at each TIS_i_ would be equal to the product of the initiation probability at TIS_i_ (P_i_) and the total number of ribosomes available to initiate at TIS_i_:

2 $$ {R}_i={P}_i\times {\displaystyle \sum_{s=i}^k{R}_s} $$

Hence, the probability of initiation at TIS_i_ (P_i_), as determined by our LS approach, is calculated as follows:

3 $$ {P}_i=\frac{R_i}{{\displaystyle \sum_{s=i}^k{R}_s}} $$

**Figure 2:**
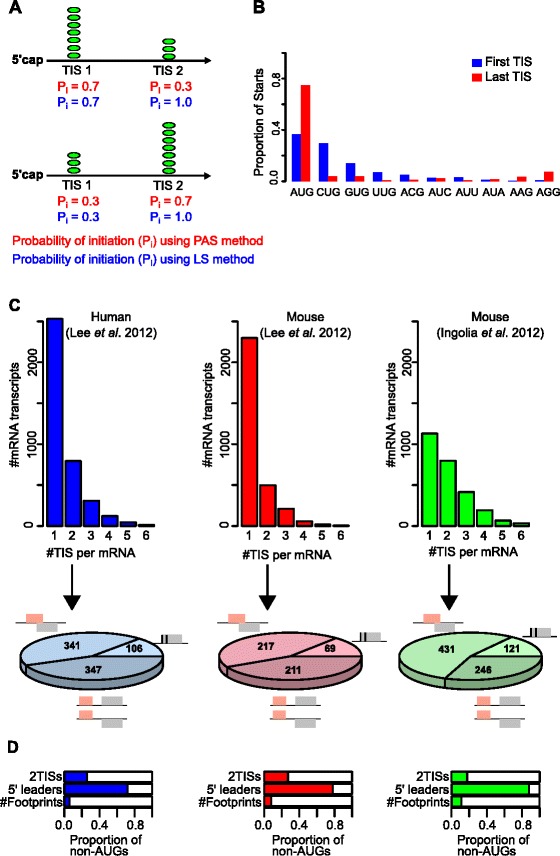
**The effect of TIS orientation on the PAS method and the LS method and selection of the data for performance comparison. A**. A schematic representation illustrating the difference in probabilities of initiation (P_i_) estimated using the PAS method (equation (1), red) and the LS method (equation (4), blue). **B**. The frequency of each codon as the first or the last TIS in an mRNA. The distributions were generated using mRNAs with multiple starts from Ingolia et al. [3] data. **C**. The barcharts indicate the number of mRNAs with a varying number of TISs (1 to 6) for genes encoding a single transcript isoform from each dataset (Lee et al. [4] (blue Human, red Mouse) and Ingolia et al. [3] (green Mouse)). The pie charts show the relative frequency of two TISs transcripts containing three different configurations of TIS ORFs: TISs that belong to overlapping ORFs (top), TISs that belong to the same ORF (middle right) and TISs that belong to ORFs separated by a non-translated region (bottom). **D**. The frequencies of non-AUG codons among all TISs (top bars) and in 5′ leaders, i.e. upstream of annotated TISs, (middle bars) in the 2 TISs mRNA datasets selected for testing the PAS and LS methods. The bottom bars “# Footprints” represent the proportion of footprint reads detected at all non-AUG TISs.

The advantage of the LS approach over the PAS approach is illustrated in Figure 2A which shows an mRNA with 2 TISs with different densities of initiating ribosomes under two conditions. When estimated with PAS, the initiation strength of TIS 2 changes according to the absolute number of ribosomes initiating there. The absolute number of ribosomes that initiate at TIS 2 is dependent on the number of ribosomes that initiated at TIS 1. Unlike with the PAS estimate, the LS estimate of TIS 2 strength is not affected by the number of ribosomes that may have initiated at the upstream TIS. The LS approach considers only the ribosomes that are available to initiate at each TIS and consequently the inferred P_i_ for each TIS_i_ is independent of the probabilities of initiation of other TISs in the same mRNA.

In this process, the density of the available scanning ribosomes is progressively reduced with each subsequent TIS. Eventually this number should fall below the detectable level so that no initiating ribosomes would be detected even for TISs with a high probability of initiation. To take this into account, we introduced an artificial TIS (designated TIS_u_ for undetectable) that represents a combination of all undetectable initiation events that take place downstream of the detectable TISs so that the mRNA now has initiation sites TIS_1_, TIS_2_, TIS_3_ … TIS_k,_ TIS_u_ and the above formula is modified as follows:

4 $$ {P}_i=\frac{R_i}{{\displaystyle \sum_{s=i}^u{R}_s}} $$

TIS_u_ represents initiation events that are undetectable with the experimental approach (green peak in Figure 1D). From our exploratory analysis, the optimum number of reads to assign to R_u_ corresponding to TIS_u_, is equal to the minimum detection threshold level used to identify a TIS (see section “Exploration of the effects of varying the 3′ artificial TIS footprint signal strength (R_u_) on the performance of the LS method”).

### Dataset for testing the LS method

Before testing the method we decided to clean the dataset to reduce falsely detected TISs. Ribosome profiling is a new technique and the nature and extent to which it could generate spurious signals is currently unknown. During our analysis we found one source of spurious signals that is best explained by the antibiotics arresting elongating ribosomes at specific codons (see [13] for independent evidence of elongating ribosome arrest at specific codons).

Due to the scanning mechanism of translation initiation, it is expected that a codon, which is inefficient as a TIS, would be detected more frequently as the first TIS (where the density of scanning ribosomes is high) than the last TIS (where the density of scanning ribosomes is reduced), as explained in Figure 1D. This was indeed the case for CUG, as can be seen in Figure 2B which shows the distribution of different codons detected as the first and the last TISs on mRNAs for the Ingolia et al. [3] data. For other non-AUG codons, the portion that was detected as the last codon increased indicating that most likely a large portion of TISs corresponding to these codons are false positives due to the arrest of elongating ribosomes (the fraction was largest for AAA which is unlikely to be used as an initiator codon, see Lee et al. [4] data in Additional file 1: Figure S1). The rule does not apply to strong AUG codons because they are expected to be the least leaky codons and hence would occur more frequently as the last TIS in a series of TISs in the same mRNA. Because the CUG set of TISs had a lower proportion of false positives, we limited the test of our method to AUG and CUG TISs only.

To observe how the LS method (equation (4)) performed in the simplest scenario, we applied it to transcripts with only 2 TISs (see Methods). To ensure that downstream initiation was due to leaky scanning, we selected TIS candidates that occur in a single frame or overlapping frames (Figure 2C,D). Thus, we excluded those cases where upon termination at the first ORF, ribosomes may resume scanning and reinitiate at a downstream codon [14-16].

### Exploration of the effects of varying the 3′ artificial TIS footprint signal strength (R_u_) on the performance of the LS method

To see how the R_u_ parameter affected the model, we varied this parameter and calculated TIS probabilities using 3 ribo-seq datasets.

When R_u_ = 0 (no artificial TIS_u_), the discrimination between AUG and CUG TISs was the highest (Additional file 1: Figure S2). This can be explained by the fact that when an artificial TIS is not included, the second TIS (i.e. the last TIS in the 2 TISs scenario) has a probability equal to 1. However, probabilities equal to 1 in the context of translation initiation is an unrealistic estimate because we cannot exclude the possibility of downstream initiation occurring below the detectable level. The minimum threshold value used for detecting a TIS in each study (0.05 R_LTM_-R_CHX_ for Lee et al. [4] data; 50 #Harr FPs for Ingolia et al. [3] data) provided the next best discrimination of AUG TISs from CUG TISs.

### Exploration of how the LS method performs in discriminating Kozak contexts

The efficiency of an initiation codon is known to depend on its nucleotide context, commonly termed the Kozak context in honour of its discoverer [17-19]. Therefore, we also examined the power of the model in discriminating TISs in favourable contexts from TISs in unfavourable contexts in transcripts with two TISs. We grouped mRNAs into three sets based on the mutual orientation of the TISs: Upstream TIS (AUG or CUG) and downstream TIS (AUG or CUG); 2. Upstream TIS (AUG) and downstream TIS (AUG); 3. Upstream TIS (CUG) and downstream TIS (AUG). To express the strength of the context numerically, we used a position specific weighting similar to previous approaches [20,21] (see Methods).

Unlike the identity of a start codon, position specific weighting scores poorly correlated with the strength of TISs irrespective of how the strength was measured (Additional file 1: Figure S3). This may be because the identity of a start codon is a larger factor affecting the strength of TISs than its context, and variation in the data does not allow our method to capture the difference. It is also possible that the read coverage in the used data is insufficient to provide the statistical power required for discriminating between codons in different Kozak contexts. To explore the latter, we carried out a characterization of the TISs using both methods under varying thresholds of read coverage. The performance of the LS method (but not the PAS method) improved as the coverage increased (Additional file 1: Figure S4).

### Exploration of the effect of scanning ribosome occlusion due to uORF translation

The LS method already takes the inhibitory effect of uTIS but not uORF translation into account, i.e. the ribosomes are sequestered at upstream TISs. Our method does not take into account the possibility that elongating ribosomes may occlude the progression of scanning ones [22,23]. The longer the ORF, the more likely trailing scanning complexes could clash with elongating ribosomes (irrespective of the relative speed of their progression), slow down and perhaps dissociate from the mRNA. Because relatively little is known about the moiety properties of scanning ribosome complexes [24], their density and the nature of their interaction with elongating ribosomes, it is impractical to model this process for the purpose of this work. Instead, we incorporated a single parameter that would take into account the inhibitory effect of elongating ribosomes on the progression of scanning complexes. The parameter represents the number of scanning ribosomes that would be lost because of a clash with elongating ribosomes and thus its value is dependent on the length of the translated ORF. We applied the approach to transcripts with 2 TISs that occur in the same frame or overlapping frames (the details are described in Additional file 1: Supplementary Text S1 and Additional file 1: Figure S5).

We found that varying the distance parameter value in the LS method (equation (4)) appeared to have a negligible effect for the 3 ribo-seq datasets examined. The predicted TISs probabilities did not improve the discrimination between non-AUG and AUG TISs (Additional file 1: Figure S6) nor correlations with the strength of Kozak context (Additional file 1: Figure S7). There may be a number of reasons for this. One possibility is the relatively short distance between TISs in the majority of transcripts examined (<100 nucleotides) and the reduction in scanning ribosomes density due to the occlusion is insignificant in comparison with the variation in the data due to the other factors [25] that we do not consider in our model. Consequently, we did not incorporate this parameter into our method for the analysis of the TISs data from the 3 ribo-seq studies analysed in this work.

### Comparison of the PAS method and the LS method for discriminating the strength of AUG TISs from non-AUG TISs

**Figure 3:**
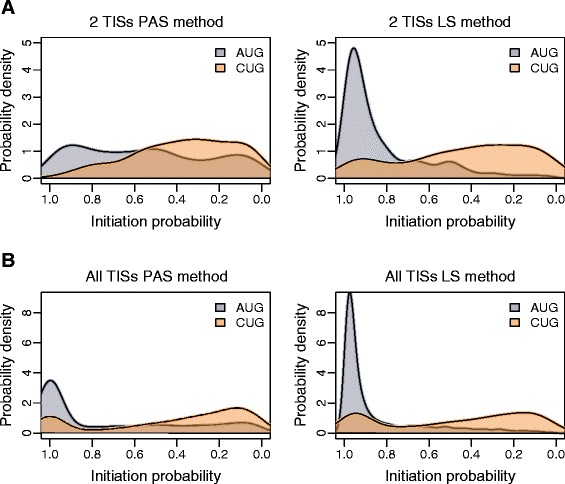
**Comparison of methods for discriminating the strength of AUG TISs from non-AUG TISs in human. A**. Probability density plots for human TISs depending on their initiation strength (gray AUG, orange CUG). Left: The scores are calculated as a fraction of the footprints aligning to the TISs from the total number of footprints aligning to the corresponding mRNA using the PAS method (equation (1)). Right: The translation initiation probability scores are calculated using the LS method (equation (4)). Transcripts with two TISs without an in-frame stop codon between the first TIS and second TIS were used. **B**. Probability density plots for human TISs from all mRNAs (left: PAS method, equation (1); right: LS method, equation (4)). All data are from Lee et al. [4] (Human) and a 3′ artificial start value of R_u_ = 0.05 R_LTM_-R_CHX_ was used for the LS method; see Additional file 1: Figure S8 for the other datasets.

Figure 3A provides the translation initiation probability density distributions for AUG and CUG TISs from the “2 TISs per mRNA human dataset” described earlier (see Additional file 1: Figure S8 for the mouse datasets). The probability densities are estimated as the relative proportion of the absolute ribo-seq signal within one mRNA using the PAS method, (equation (1), left) and using the LS method (equation (4), right). We also applied the two methods to all mRNAs with any number of TISs, including those that contain non-overlapping ORFs, thus allowing for the possibility of reinitiation (Figure 3B). It is evident that that LS method (equation (4)) discriminates initiation probabilities at AUG and CUG codons much better and this argues for its superiority over the PAS method (equation (1)).

Interestingly, the discriminatory power of the LS method over the PAS method improved with increased footprint coverage (Additional file 1: Figure S9). However, while the separation of AUG and CUG codons clearly improved, it is also clear that the distribution of TISs was also affected as CUG codons with low probability were almost entirely eliminated from the higher footprint coverage distributions (Additional file 1: Figure S9).

Nevertheless, it is apparent from this analysis that AUG codons on average are much stronger initiators than CUG codons. In the 3919 single isoform transcripts for which AUG/CUG TISs were detected, we found that in the 5′ leaders, defined as the region between the 5′ end of an mRNA and the most 3′ TIS, there are 23005 AUG codons and 41186 CUG codons. Of these, 20% of AUG codons produced a detectable translation initiation signal, while only 3% of CUG codons were detected as TISs.

## Discussion

It is apparently clear that translation initiation in many mammalian mRNAs takes place at more than one location. Multiple TISs play different functions. TISs that are in-frame with the main protein coding ORF allow various protein isoforms to be synthesized. Translation of short ORFs from TISs that do not belong to the main coding ORFs provides mechanisms for responding to various changes in physiological conditions (see [26] for a review). Approximately 35-40% of mammalian mRNAs are estimated to possess uAUG codons [20]. Translation initiation, however, is not limited to AUG codons. Many non-AUG coded N-terminal extensions are evolutionarily conserved [9] and some of these variants play vital functions, e.g. the recently discovered CUG initiated variant of PTEN [27]. Ribosome profiling studies described in this work [3,4] provide evidence that the frequency of non-AUG TISs could be as high as that of AUG TISs. Therefore 40% of mRNAs with multiple TISs seems to be an underestimate. This multiplicity of TISs is not reflected in the major bioinformatics resources, where coding sequences in mRNAs are usually annotated with a single TIS. To overcome this problem, Wan and Qian developed TISdb [28], a database of TISs detected with ribosome profiling carried out in human and mouse. While the information in the database is very useful, binary classification of codons as TISs or non-TISs has serious limitations. Assigning strength scores to TISs could help the interpretation of their individual functional role in the translational control of their mRNAs. Therefore we have developed a computational approach for converting the translation initiation signal obtained with ribosome profiling into probabilities of ribosome initiation at individual TISs.

## Conclusions

The application of this method to two types of TISs (AUG and CUG) allowed us to conclude that AUG and CUG codons have markedly different properties as translation initiators. We corroborated earlier observations that AUG TISs on average are much stronger initiators than CUG TISs. As can be seen in Figure 3, the majority of AUG TISs are very good initiators with the probability of translation initiation being close to 1. In addition there are weak AUG codons that cover a wide range of initiation probabilities. Perhaps, these codons are located in highly unfavourable contexts for initiation or there are specific factors that inhibit initiation at these codons. The behaviour of CUG codons is the opposite: the majority of CUG codons are very poor initiators, while a minority of CUG codons exhibit strong translation initiation (though see Results for the effect of sequence coverage on the distribution). Unlike weak AUG codons, perhaps these strong CUG codons are located in highly favourable initiation contexts or there are specific factors that activate initiation at these codons. The majority of detectable CUG TISs occur in mRNA 5′ leaders where the density of scanning ribosomes is the highest. At these locations even infrequent initiation could produce a significant signal. Therefore CUG TISs can be responsible for significant protein/peptide production from highly expressed mRNAs if they are located upstream of AUG TISs, despite their weakness as initiators. Indeed, many short open reading frames (sORFs) encoding sORF-encoded polypeptide (SEP) products have been found to initiate with non-AUG start codons [29,30]. However it has been reported that despite the large scale detection of alternative translation initiation in recent proteomics studies (with matching ribo-seq data), only a few mass spectrometry detectable translation initiation events occurred at non-AUG codons [31,32]. Future ribosome profiling of initiating ribosome studies, in particular the QTI-seq approach (the quantitative profiling of initiating ribosomes developed by the Qian lab, Shu-Bing Qian personal communication, Recoding Meeting, Killarney, Ireland, 13–18 May 2014) will undoubtedly provide better data which in turn will likely improve the accuracy of the initiation probability scores as determined by the LS method.

In addition to resolving the contradiction caused by the observation of a large number of non-AUG TISs in ribo-seq studies, the LS approach will be useful for the interpretation of future ribo-seq data. It should help in the understanding of how translation initiation is regulated in each specific mRNA in response to various conditions as well as the characterization of the features of start codon selection which are specific to a particular tissue or developmental stage. When an mRNA has multiple TISs, the absolute number of ribosomes initiating at a particular TIS depends on the absolute number of ribosomes initiating at other (upstream) TISs for the same mRNA (Figure 2A). The probabilities of initiation at the TISs, however, are independent of each other. Therefore determining the probabilities of initiation at different TISs is more informative for finding TISs that are under regulation than looking at the changes in the absolute number of initiating ribosomes. For instance, the down regulation of the main ORF translation may happen as a consequence of uORF translation up regulation. But how can this be determined? If the regulation is mediated through the uORF TIS and not the main ORF TIS, a change in the probability of uORF initiation would be expected, but not of the main ORF, while the absolute levels will change for both (Figure 2A). Consequently, estimating the probability of ribosomes initiating at a start codon is more informative.

## Methods

The ribosome profiling data of initiating ribosomes used in our analysis were obtained from Supplementary Tables sd01.xlsx and sd03.xlsx from Lee et al. [4] and Supplementary Table S3 Sites of Translation Initiation from Ingolia et al. [3].

The “All TISs” distributions in Figure 1A were calculated using the frequency of each TIS type in the Lee et al. [4] tables and in Ingolia et al. [3] table. The “5′ leaders” distributions in Figure 1A were calculated using the frequency of the TISs identified as occurring upstream of the annotated TIS (aTIS) in the Lee et al. [4] tables and in the Ingolia et al. [3] table. The “# Footprints” distributions in Figure 1A were calculated for AUG codons and codons that are near-cognate to AUG (i.e. codons that differ from AUG by a single nucleotide) using the absolute number of footprint reads obtained under each antibiotic treatment (“LTM reads” in Tables sd01.xlsx andsd03.xlsx from Lee et al. [4] and “# Harr Reads” in Supplementary Table S3 from Ingolia et al. [3].

For panels B and C of Figure 1, the R_LTM_-R_CHX_ field in Supplementary Tables sd01.xlsx and sd03.xlsx was used for the Lee et al. [4] data while the “# Harr Reads” field in Supplementary Table S3 was used for the Ingolia et al. [3] data.

Lee et al. [4] describe R_LTM_-R_CHX_ as R_k_ = (X_k_/N_k_) × 10 (k = LTM, CHX), where X_k_ is the number of reads corresponding to the TIS in data k, and N_k_ is the total number of reads corresponding to the TIS mRNA in data k. Lee et al. [4] used 0.05 R_LTM_-R_CHX_ as the minimum threshold value for TIS detection. The minimum number of #Harr FPs in Supplementary Table S3 from Ingolia et al. [3] is 50. Unless otherwise specified, the data from these fields respectively were used in all applications of the PAS and LS methods for estimating the probabilities of initiation as described in Results.

### Selecting candidates for testing the LS method

Transcripts with multiple TISs were used for the calculation of the frequency of each codon start type as the first or the last TIS in an mRNA for Figure 2B and Additional file 1: Figure S1. As these figures suggest that elongating ribosomes may have been captured at many of the non-AUG codon types, we considered AUG and CUG start codons only for the application of our methods.

Two or more transcript variants of the same gene may share the same start site(s) assignations. As our approach is based on the leaky scanning model of translation initiation, we wished to ensure that the footprint data for 2 or more TISs originate from the same mRNA transcript. Consequently, we considered genes that have only one transcript isoform according to the annotations provided in Supplementary Tables sd01.xlsx and sd03.xlsx from Lee et al. [4] and Supplementary Table S3 Sites of Translation Initiation from Ingolia et al. [3] (the distribution of the number of TISs across the single isoform genes are provided as barcharts in Figure 2C).

We applied the PAS method (equation (1)) and the LS method (equation (4)) (see Results section) to single isoform genes with two detected TISs (Figure 3A). To ensure that initiation at the downstream TIS was solely a result of leaky scanning, we only considered TIS candidates where there was no in-frame stop codon between the first TIS and second TIS to exclude the possibility of reinitiation (the number of candidates is provided in the pie charts in Figure 2C). For Figure 3B, single isoform transcripts with one or more TISs, where the start codon types were either AUG or CUG, were considered. In addition, we did not apply filters that eliminate ORF configurations that allow reinitiation.

For Figure 3 where the LS method (equation (4)) was applied, the minimum footprint threshold value used for detecting a TIS in each study (0.05 R_LTM_-R_CHX_ for Lee et al. [4] data and #50 Harr FPs for Ingolia et al. [3] data) was assigned to each 3′ artificial start parameter, R_u_, in each mRNA.

### Position specific weight matrices for Kozak context

To discriminate between different Kozak contexts, a position specific weight matrix for each dataset was generated for the 3 nucleotides upstream of each TIS (positions −3, −2, −1) and the first nucleotide position downstream of each TIS (position +4) (the first nucleotide of the TIS is considered as position +1). The position specific frequency for each nucleotide was calculated using the contexts of the TISs considered (AUG and CUG TISs in transcripts with 2 TISs and no in-frame stop codon between starts). These frequencies were used to assign weightings to each nucleotide in each position of the context. For a given TIS, a Kozak context score was calculated based on the sum of the position specific weightings for the context of the TIS. Typically, ACC - - - G has the highest context score and TTT - - - T the lowest context score.

### The LS TIS probability scores are available in GWIPS-viz

The probabilities of translation initiation at TISs in single transcript isoform genes described in this work are available as a GWIPS-viz browser [33] track at http://gwips.ucc.ie where it can be explored in conjunction with other ribosome profiling data (Additional file 1: Figure S10). The probability scores are available to download from the “Tables” tab in GWIPS-viz.

## Additional file

Additional file 1:
**Supplementary Text S1 and Figures S1-S10.**

## Abbreviations

ribo-seq: Ribosome profiling
TIS: Translation initiation site
aTIS: Annotated translation initiation site
uTIS: Upstream translation initiation site
dTIS: Downstream translation initiation site
PAS: Proportion of the absolute signal
LS: Leaky scanning

## References

[1] Ingolia NT, Ghaemmaghami S, Newman JRS, Weissman JS (2009). Genome-wide analysis in vivo of translation with nucleotide resolution using ribosome profiling. Science.

[2] Michel AM, Baranov PV (2013). Ribosome profiling: a Hi-Def monitor for protein synthesis at the genome-wide scale. Wiley Interdiscip Rev RNA.

[3] Ingolia NT, Lareau LF, Weissman JS (2011). Ribosome profiling of mouse embryonic stem cells reveals the complexity and dynamics of mammalian proteomes. Cell.

[4] Lee S, Liu B, Lee S, Huang S-X, Shen B, Qian S-B (2012). Global mapping of translation initiation sites in mammalian cells at single-nucleotide resolution. ProcNatlAcad Sci U S A.

[5] Fritsch C, Herrmann A, Nothnagel M, Szafranski K, Huse K, Schumann F, Schreiber S, Platzer M, Krawczak M, Hampe J, Brosch M (2012). Genome-wide search for novel human uORFs and N-terminal protein extensions using ribosomal footprinting. Genome Res.

[6] Anderson CW, Buzash-Pollert E (1985). Can ACG serve as an initiation codon for protein synthesis in eucaryoticcells?. Mol Cell Biol.

[7] Hann SR, King MW, Bentley DL, Anderson CW, Eisenman RN (1988). A non-AUG translational initiation in c-myc exon 1 generates an N-terminally distinct protein whose synthesis is disrupted in Burkitt’slymphomas. Cell.

[8] Peabody DS (1989). Translation initiation at non-AUG triplets in mammalian cells. J Biol Chem.

[9] Ivanov IP, Firth AE, Michel AM, Atkins JF, Baranov PV (2011). Identification of evolutionarily conserved non-AUG-initiated N-terminal extensions in human coding sequences. Nucleic Acids Res.

[10] Kozak M (1989). Context effects and inefficient initiation at non-AUG codons in eucaryotic cell-free translation systems. Mol Cell Biol.

[11] Clements JM, Laz TM, Sherman F (1988). Efficiency of translation initiation by non-AUG codons in saccharomyces cerevisiae. Mol Cell Biol.

[12] Kozak M (2002). Pushing the limits of the scanning mechanism for initiation of translation. Gene.

[13] Dmitriev SE, Akulich KA, Andreev DE, Terenin IM, Shastky IN (2013). The peculiar mode of translation elongation inhibition by antitumor drug harringtonin. FEBS J.

[14] Kozak M (1987). Effects of intercistronic length on the efficiency of reinitiation by eucaryoticribosomes. Mol Cell Biol.

[15] Pöyry TAA, Kaminski A, Jackson RJ (2004). What determines whether mammalian ribosomes resume scanning after translation of a short upstream open reading frame?. Genes Dev.

[16] Hinnebusch A (2005). Translational regulation of GCN4 and the general amino acid control of yeast. Annu Rev Microbiol.

[17] Kozak M (1986). Point mutations define a sequence flanking the AUG initiator codon that modulates translation by eukaryotic ribosomes. Cell.

[18] Kozak M (1987). An analysis of 5′-noncoding sequences from 699 vertebrate messenger RNAs. Nucleic Acids Res.

[19] Kozak M (1997). Recognition of AUG and alternative initiator codons is augmented by G in position +4 but is not generally affected by the nucleotides in positions +5 and +6. EMBO J.

[20] Rogozin IB, Kochetov AV, Kondrashov A, Koonin EV, Milanesi L (2001). Presence of ATG triplets in 5′ untranslated regions of eukaryotic cDNAs correlates with a “weak” context of the start codon. Bioinformatics.

[21] Nakagawa S, Niimura Y, Gojobori T, Tanaka H, Miura K (2008). Diversity of preferred nucleotide sequences around the translation initiation codon in eukaryote genomes. Nucleic Acids Res.

[22] Kozak M (1995). Adherence to the first-AUG rule when a second AUG codon follows closely upon the first. Proc Natl Acad Sci U S A.

[23] Kozak M (2001). Constraints on reinitiation of translation in mammals. Nucleic Acids Res.

[24] Vassilenko KS, Alekhina OM, Dmitriev SE, Shatsky IN, Spirin AS (2011). Unidirectional constant rate motion of the ribosomal scanning particle during eukaryotic translation initiation. Nucleic Acids Res.

[25] Zur H, Tuller T (2013). New universal rules of eukaryotic translation initiation fidelity. PLoS Comput Biol.

[26] Liu B, Qian S-B (2013). Translational reprogramming in cellular stress response. Wiley Interdiscip Rev RNA.

[27] Hopkins BD, Fine B, Steinbach N, Dendy M, Rapp Z, Shaw J, Pappas K, Yu JS, Hodakoski C, Mense S, Klein J, Pegno S, Sulis M-L, Goldstein H, Amendolara B, Lei L, Maurer M, Bruce J, Canoll P, Hibshoosh H, Parsons R (2013). A secreted PTEN phosphatase that enters cells to alter signaling and survival. Science.

[28] Wan J, Qian S-B (2014). TISdb: a database for alternative translation initiation in mammalian cells. Nucleic Acids Res.

[29] Slavoff SA, Mitchell AJ, Schwaid AG, Cabili MN, Ma J, Levin JZ, Karger AD, Budnik BA, Rinn JL, Saghatelian A (2013). Peptidomic discovery of short open reading frame-encoded peptides in human cells. Nat Chem Biol.

[30] Ma J, Ward CC, Jungreis I, Slavoff SA, Schwaid AG, Neveu J, Budnik BA, Kellis M, Saghatelian A (2014). Discovery of human sORF-encoded polypeptides (SEPs) in cell lines and tissue. J Proteome Res.

[31] Menschaert G, Van Criekinge W, Notelaers T, Koch A, Crappé J, Gevaert K, Van Damme P (2013). Deep proteome coverage based on ribosome profiling aids mass spectrometry-based protein and peptide discovery and provides evidence of alternative translation products and near-cognate translation initiation events. Mol Cell Proteomics.

[32] Van Damme P, Gawron D, Van Criekinge W, Menschaert G (2014). N-terminal proteomics and ribosome profiling provide a comprehensive view of the alternative translation initiation landscape in mice and men. Mol Cell Proteomics.

[33] Michel AM, Fox G, M Kiran A, De Bo C, O’Connor PBF, Heaphy SM, Mullan JPA, Donohue CA, Higgins DG, Baranov PV (2014). GWIPS-viz: development of a ribo-seq genome browser. Nucleic Acids Res.

