# Supplementary material for: Computational approach for calculating the probability of eukaryotic translation initiation from ribo-seq data that takes into account leaky scanning
Source: BMC Bioinformatics. 2014 Nov 21;15(1):380. doi: 10.1186/s12859-014-0380-4 (PMC4245810; doi:10.1186/s12859-014-0380-4)
Supplement: Additional file 1: — Supplementary Text S1 and Figures S1-S10. [file 12859_2014_380_MOESM1_ESM.pdf]

Supplementary Text S1: Exploration of the effects of varying the distance parameter (k) on the performance of the LS approach.

We wished to incorporate the inhibitory effect of ORF length on downstream initiation into our LS method (equation (4)) in Results, main text) for TISs that belong to overlapping ORFs and TISs that belong to the same ORF. Our strategy was to incorporate artificial distance starts, TIS_{Di}, between TISs in an mRNA with TIS_1, TIS_2, TIS_3 ... TIS_k, TIS_u, as follows:

TIS_1, TIS_{D1}, TIS_2, TIS_{D2}, TIS_3, TIS_{D3},... TIS_k, TIS_u **(5)** (following on from equation (4) in Results, main text)

where artificial starts TIS_{Di} represent scanning ribosomes that have disassociated between TIS_i and TIS_{i+1} and D_i represents the nucleotide distance between the TIS_i and TIS_{i+1}.

The probability of ribosomes disassociating between TIS_i and TIS_{i+1} should positively correlate with the distance D_i (the longer the ORF of TIS_i, the more likely scanning ribosomes will encounter elongating ribosomes and be forced to disassociate from the mRNA before reaching TIS_{i+1}).

The probability of scanning ribosomes disassociating should also correlate positively with the number of footprint reads for TIS_i: the more ribosomes that initiate at TIS_i, the higher the density of elongating ribosomes between TIS_i and TIS_{i+1}, and consequently, the more likely scanning ribosomes will encounter elongating ribosomes and disassociate from the mRNA.

Hence, the probability of disassociation, P_{di}, at TIS_{Di} should approach 1 when D_i and R_i (absolute number of footprint reads at TIS_i) approach infinity. Likewise, the probability of disassociation P_{di}, should approach 0 when D_i and R_i approach 0 (there can be no loss of scanning ribosomes if no ribosomes initiate at TIS_i). We propose the following function which satisfies the above criteria:

$$P_{di} = 1 - k^{DiRi} \quad \textbf{(6)}$$

where k is a parameter that can range from 0 to 1. Note that k=1 is equivalent to not taking the distance between TISs into account. A suitable value for k can be determined by fitting different values for k to the data. However, in order to do this, the number of disassociated scanning ribosomes (R_{di}) for each artificial distance start (TIS_{Di}) needs to be incorporated into our approach.

The number of disassociated scanning ribosomes is equal to the product of the probability of scanning ribosomes disassociating and the number of available ribosomes:

$$R_{d\,i} = P_{di} \times \sum_{s=Di}^{u} R_s \qquad \textbf{(7)}$$

where s starts from TIS_{Di} (does not include footprint reads from the previous TIS_i).

We do not know the number of scanning ribosomes that can potentially disassociate at each artificial distance start TIS_{Di}. However, we can use the absolute number of footprints detected at each TIS_i in the data to express R_{di}. The simplest scenario of two TISs (TIS_1, TIS_2), with artificial distance start TIS_{D1} and 3'artificial start TIS_u will be used to illustrate the estimation of R_{d1}. From (7),

$$R_{d1} = P_{d1}(R_{d1} + R_2 + R_u) \qquad \textbf{(8)}$$

$$\frac{R_{d1}}{P_{d1}} = R_{d1} + R_2 + R_u \qquad \textbf{(9)}$$

$$\frac{R_{d1}}{P_{d1}} - R_{d1} = R_2 + R_u \qquad \textbf{(10)}$$

$$R_{d1} - R_{d1}P_{d1} = P_{d1}(R_2 + R_u) \qquad \textbf{(11)}$$

$$R_{d1}(1 - P_{d1}) = P_{d1}(R_2 + R_u) \qquad \textbf{(12)}$$

$$R_{d1} = \frac{P_{d1}(R_2 + R_u)}{(1 - P_{d1})} \qquad \textbf{(13)}$$

Substituting $1\text{-}k^{DiRi}$ for P_{di} from (6), we get

$$R_{d1} = \frac{(1 - k^{D1R1})(R_2 + R_u)}{k^{D1R1}} \qquad \textbf{(14)}$$

Extending this to the general case:

$$R_{di} = \frac{(1 - k^{DiRi})\sum_{s=i+1}^{u} R_s}{k^{DiRi}} \qquad \textbf{(15)}$$

Having an estimation (using the number of actual footprint reads at each detected TIS) for the number of disassociated scanning ribosomes R_{di} for each TIS_{Di}, we can then calculate the probabilities P_i for each TIS_i in an mRNA using our LS method (equation (4) in Results, main text), but now include the estimated number of disassociated ribosomes R_{di} for each artificial distance start TIS_{Di} for an mRNA with TIS_1, TIS_{D1}, TIS_2, TIS_{D2}, TIS_3, TIS_{D3},... TIS_k, TIS_u.

The question remains as to a suitable value for k? To estimate k for the different datasets, we used single isoform transcripts with 2 TISs and no in-frame stop codon between TIS_1 and TIS_2, and R_u (3' artificial TIS) equal to the minimum TIS detection threshold used in each study (Methods). We generated simple linear regressions of the ratios P_1/P_2 (for different values of k), regressed onto the corresponding nucleotide distances between TIS_1 and TIS_2 for the transcripts considered. We compared these regression slopes (blue plots in Supplementary Figure S6) with the slopes obtained from regressing P_1/P_2 onto the distances between TIS_1 and TIS_2 where distance is not accounted for (equivalent to k=1) (red plots in Supplementary Figure S6).

The motivation and assumptions for this are explained below:

1. We assume that the further TIS_2 is from TIS_1, the more scanning ribosomes are lost, which should result in an overestimation of P_1 and an underestimation of P_2. That is, P_1/P_2 increases with distance (positive upward slope) (see slopes in the red plots in Supplementary Figure S6).

2. We assume that TISs of different strengths are distributed randomly in the mRNA.

3. If probabilities are estimated accurately there should be no correlation between the probability of initiation at the second codon and the distance between TIS_1 and TIS_2.This suggests that if the distance factor is correctly accounted for, the slope of the curve of P_1/P_2 ratios regressed onto the distances between TIS_1 and TIS_2, should become close to 0.

The following values for k were found to redress the slopes nearer to zero (slopes in blue plots compared to the slopes in the red plots): Human (Lee *et al.* [4] data) k= 0.999; Mouse (Lee *et al.* [4] data) k =0.995; and Mouse (Ingolia *et al.* [3] data) k= 0.99999.

The corresponding probability distribution plots using these values of k (Supplementary Figure S6), however, do not show any improvement in discriminating the strength of initiation of AUG TISs from CUG TISs compared to when the distance between TISs is not taken into account (equivalent to k=1).

Exploration of the effects of varying the distance parameter k on Kozak context discrimination.

The effect of accounting for the distances between TISs and discrimination of Kozak contexts was investigated. However, as can be seen in Supplementary Figure S7, incorporating a distance factor had little effect for the 3 ribo-seq datasets analysed. The slopes of the regression curves when the distance between TISs is not considered (equivalent to k=1) (blue plots) are steeper compared to the slopes for lower values of the distance parameter k (green plots with different values for k).

Nevertheless, our LS method with a default distance parameter value of k=1 provides better discrimination of Kozak contexts compared to the regression slopes obtained when Kozak context scores are regressed onto the probabilities obtained using the PAS method (red plots).

Supplementary Figures

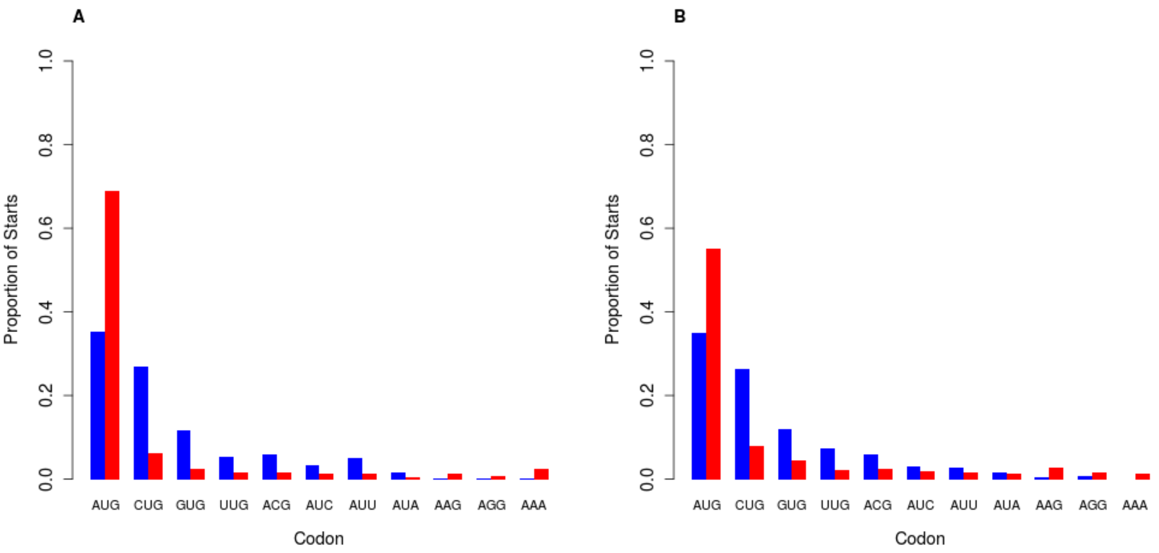

Figure S1. The frequency of each codon as the first or the last TIS in an mRNA. The distributions were generated using Lee *et al.* [4] data (Panel A, Human; Panel B, Mouse).

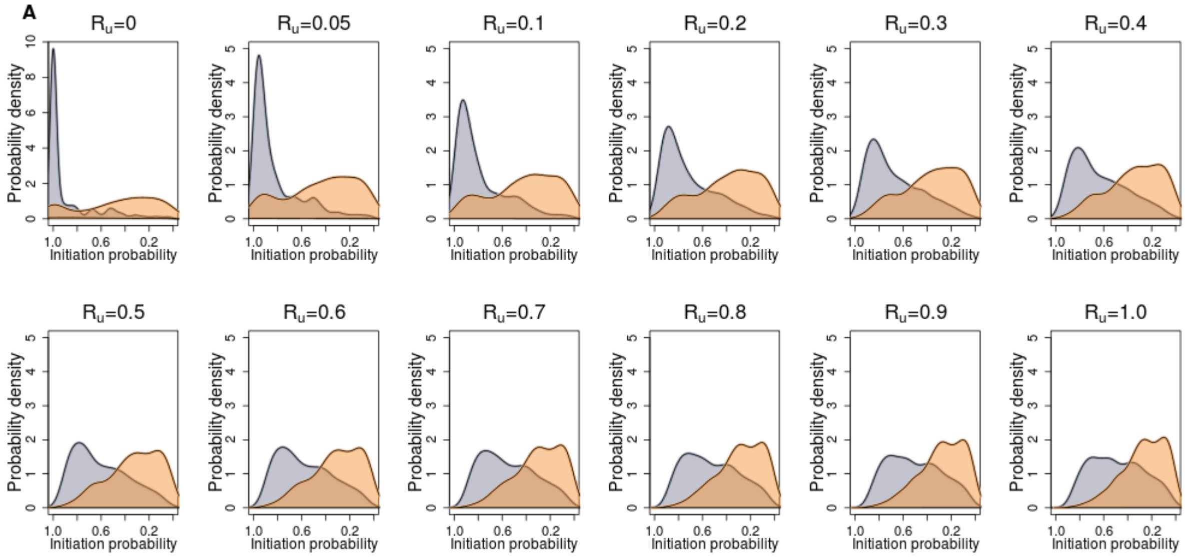

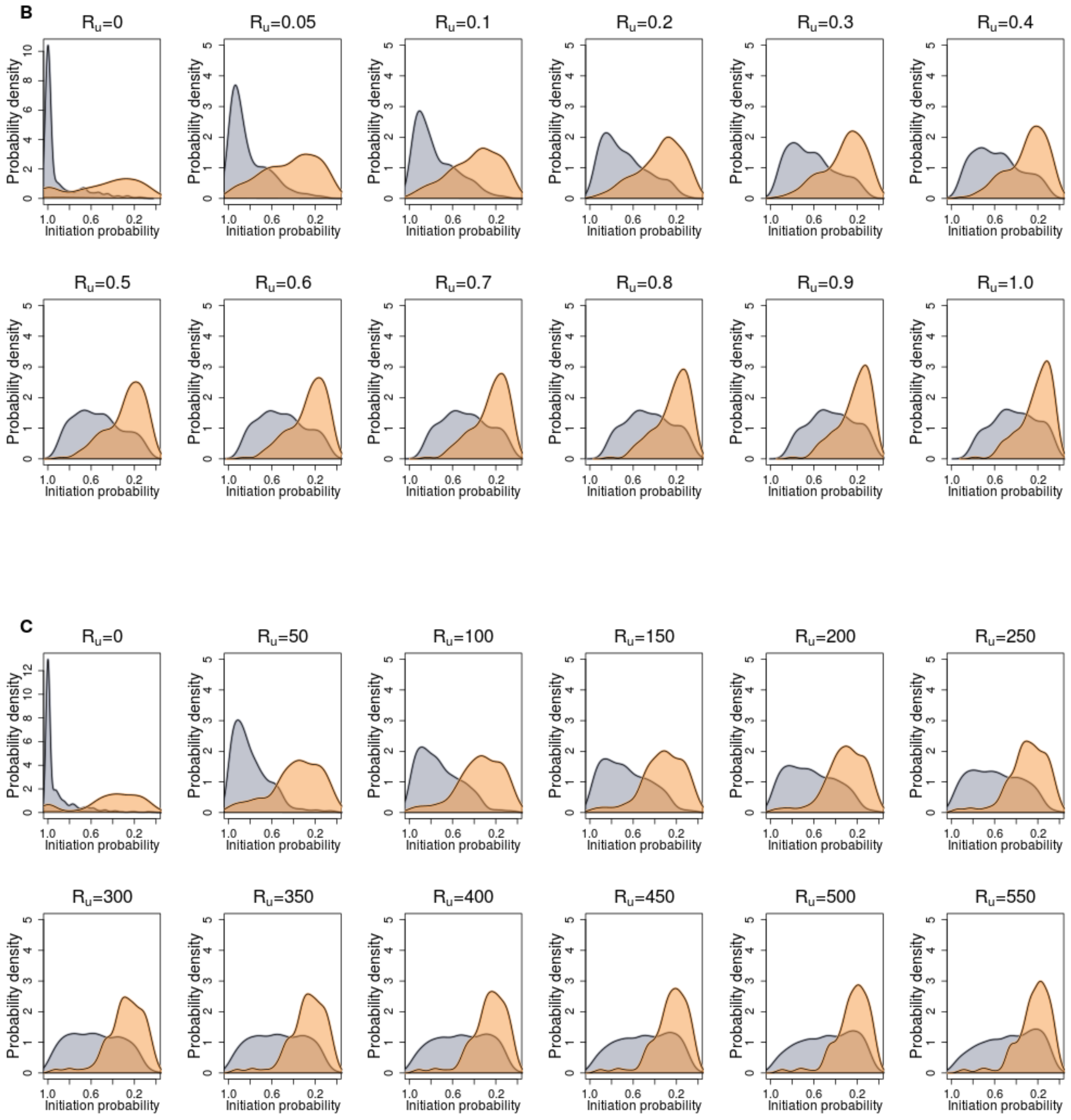

Figure S2. The distributions of translation initiation probability scores (represented as kernel density plots) for AUG (grey) and CUG (orange) TISs when the R_u parameter was varied for transcripts with two TISs with no in-frame stop codon between the two TISs. The distributions were generated using data from Lee *et al.* [4] (A Human, B Mouse) and Ingolia *et al.* [3] (C Mouse).

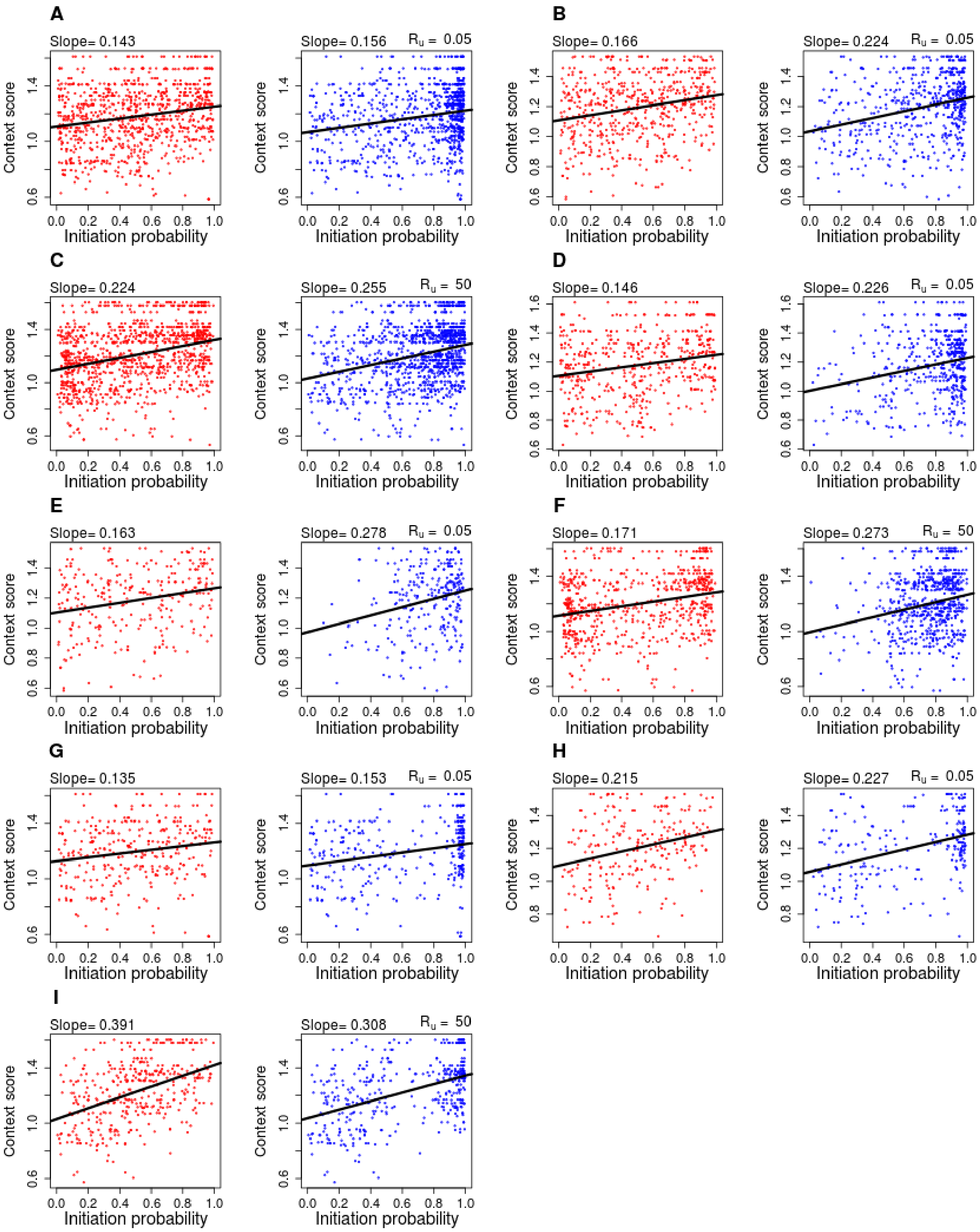

Figure S3. Exploration of how the LS method performs in discriminating Kozak contexts. **A.** The slopes when Kozak context scores are regressed onto the probability scores generated for Human (Lee *et al.* [4] data). The two TISs transcript dataset with AUG and CUG codons

described previously was used. The red plot gives the slope of the curve when the Kozak context scores are plotted against the proportion of footprints for a TIS from the total number of footprints for the mRNA (PAS method, equation (1) in Results, main text). The blue plot shows the regression slope when the LS approach (equation (4) in Results, main text) is applied using a 3' artificial start value of R_u=0.05 R_{LTM}-R_{CHX}. **B.** Generated for Mouse (Lee *et al.* [4] data) with the same description as panel A. **C**. Generated for Mouse (Ingolia *et al.* [3] data) with the same description as panel A except that a 3' artificial start value of R_u=50 #Harr FPs was used. **D,E,F.** same as A,B,C respectively except that transcripts with two AUG TISs were used. **G,H,I.** same as A,B,C respectively except that transcripts with an upstream CUG TIS and a downstream AUG TIS were used.

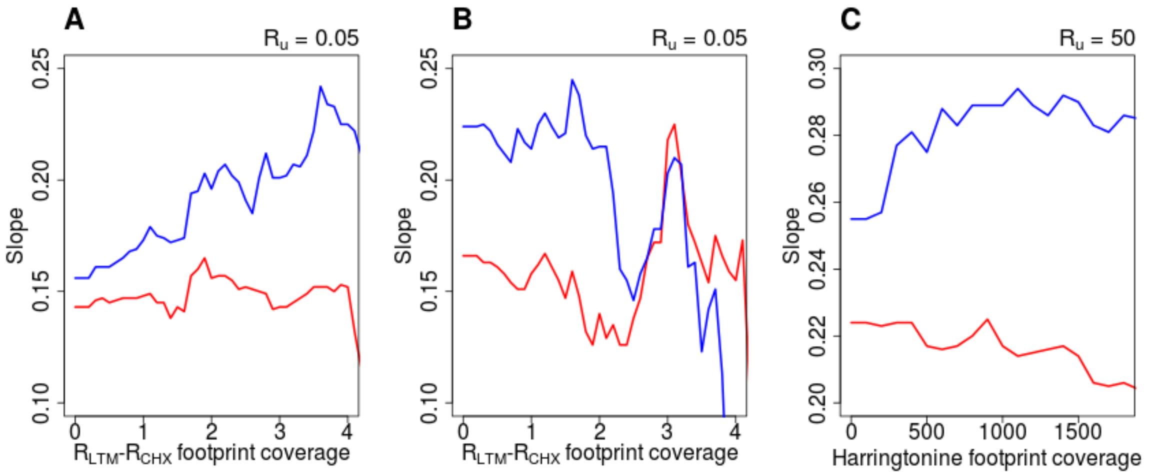

Figure S4 Exploration of how the PAS and LS methods perform in discriminating Kozak contexts for different footprint coverage thresholds (i.e. the cumulative footprint coverage for the 2TISs in the mRNA). **A.** The slope values for different cumulative footprint coverage thresholds when Kozak context scores are regressed onto the probability scores generated for Human (Lee *et al.* [4] data). The two TISs transcript dataset with AUG and CUG codons described previously was used. The red line represents the slope values when the Kozak context scores are plotted against the proportion of footprints for a TIS from the total number of footprints for the mRNA (PAS method, equation (1) in Results, main text) for different cumulative R_{LTM}-R_{CHX} footprint coverage thresholds. The blue line shows the

different slope values when the LS approach (equation (4) in Results, main text) is applied. A 3' artificial start value of R_u=0.05 R_{LTM}-R_{CHX} was used. **B.** Generated for Mouse (Lee *et al.* [4] data) with the same description as panel A. **C**. Generated for Mouse (Ingolia *et al.* [3] data) with the same description as panel A except for different cumulative #Harringtonine footprint coverage thresholds and a 3' artificial start value of R_u=50 #Harr FPs.

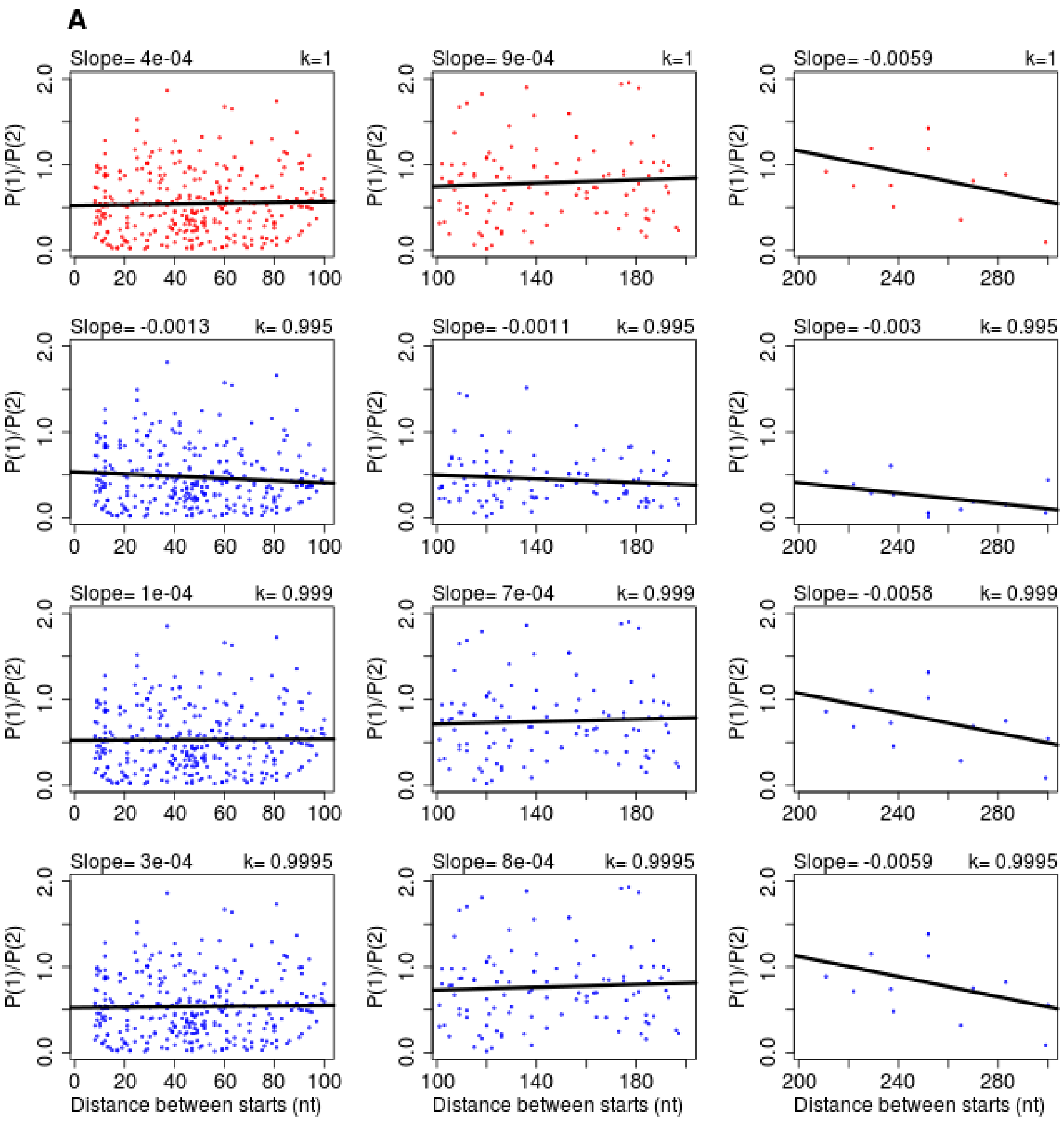

B

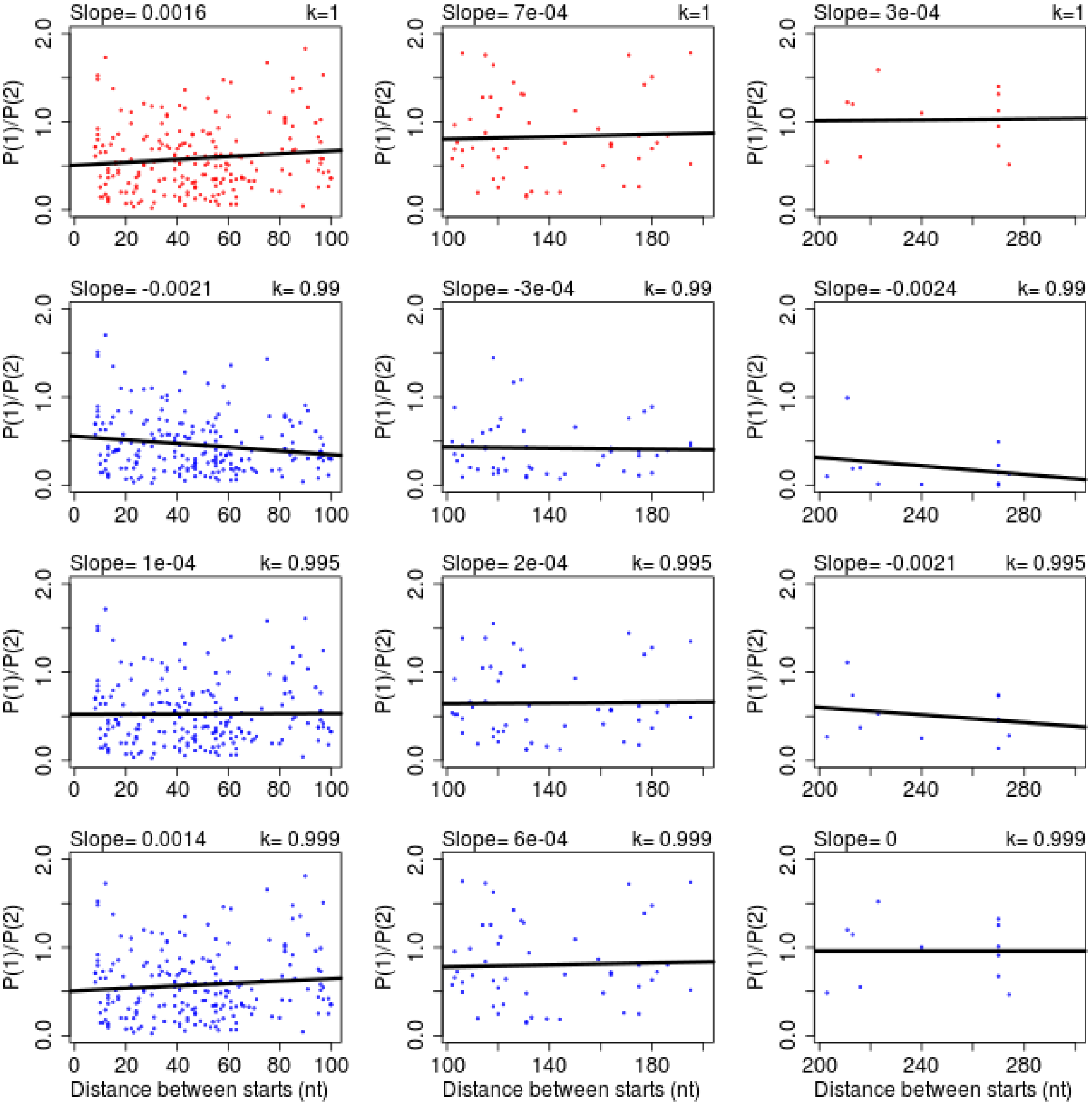
Slope= 0.0016
k=1
Slope= 7e-04
k=1
Slope= 3e-04
k=1
Slope= -0.0021
k= 0.99
Slope= -3e-04
k= 0.99
Slope= -0.0024
k= 0.99
Slope= 1e-04
k= 0.995
Slope= 2e-04
k= 0.995
Slope= -0.0021
k= 0.995
Slope= 0.0014
k= 0.999
Slope= 6e-04
k= 0.999
Slope= 0
k= 0.999
P(1)/P(2)
Distance between starts (nt)

C

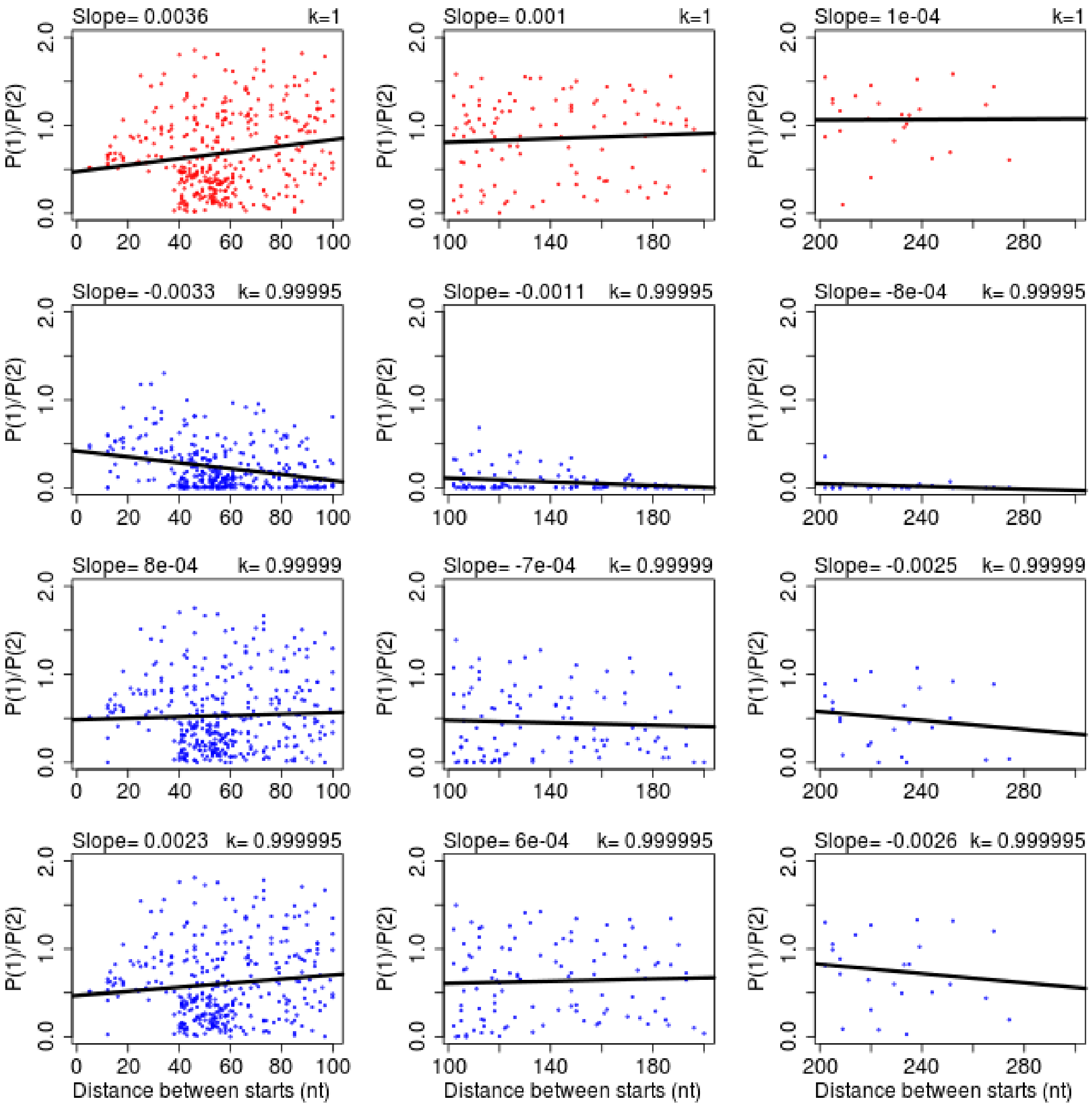

Figure S5. Regression curves to determine the optimum value for the distance factor k. **A.** The red plots provide the slopes obtained from regressing P_1/P_2 onto the distances between TIS_1 and TIS_2 where distance is not accounted for (equivalent to k=1). The blue plots provide the slopes when the ratios P_1/P_2 obtained with different values for k, are regressed onto the distances between starts. The distances are in bins of 100 nucleotides. The plots are generated for Human (Lee *et al.* [4] data) and a 3' artificial start value of R_u=0.05 R_{LTM}-R_{CHX} was used. **B.** Generated for Mouse (Lee *et al.* [4] data) with the same description as panel A. **C**. Generated for Mouse (Ingolia *et al.* [3] data) with the same description as panel A except that a 3' artificial start value of R_u=50 #Harr FPs was used.

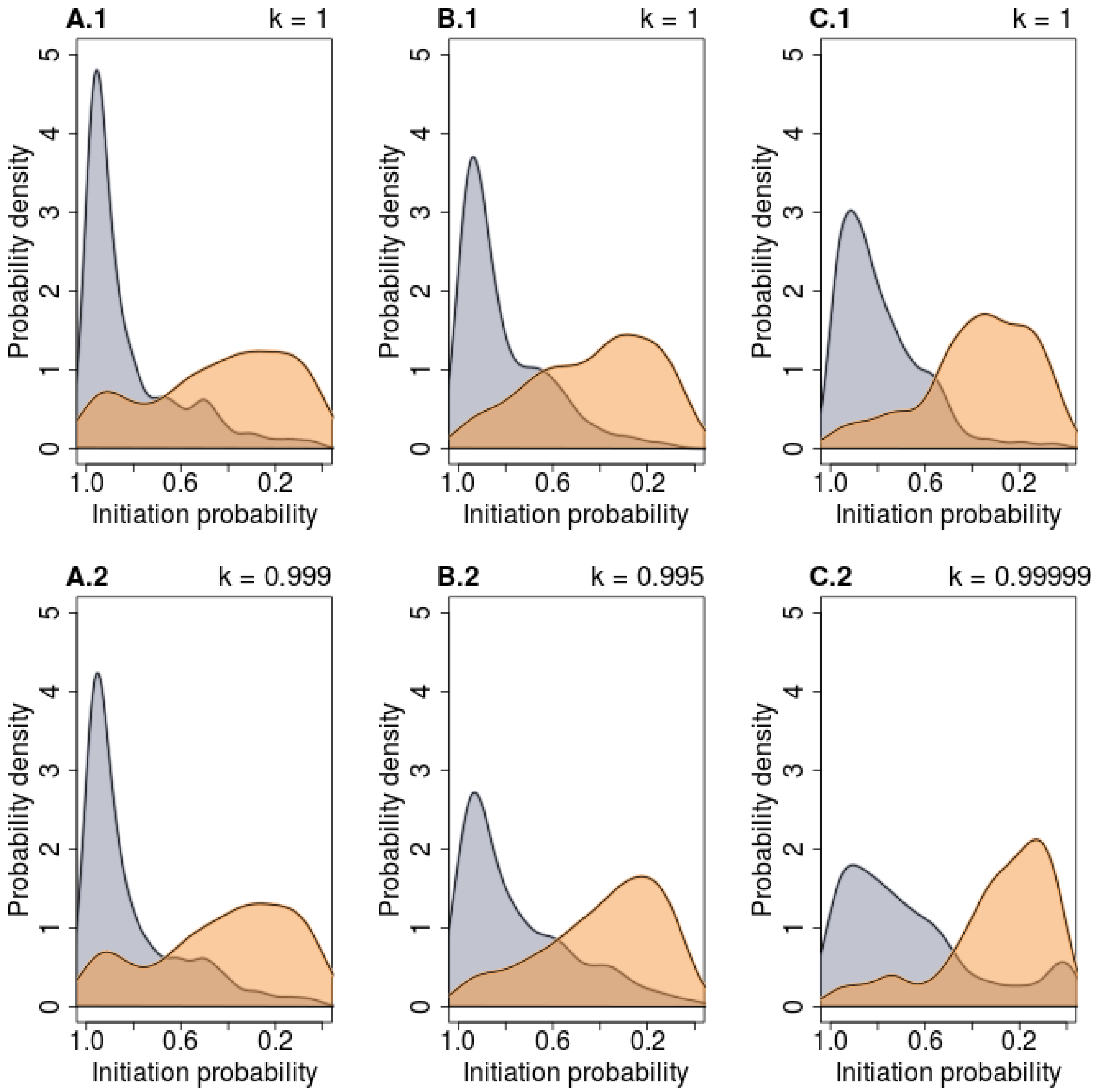

Figure S6. Performance of the LS method in discriminating the probabilities of translation initiation at AUG TISs from CUG TISs when the distance between TISs is not accounted for (top panel) and when a distance factor is incorporated into the method (bottom panel). The distributions of probability scores for individual TISs (gray AUG, orange CUG) are represented as kernel density plots. Figures are generated using initiating ribosome footprint data from Lee *et al.* [4] (A Human, B Mouse) and Ingolia *et al.* [3] (C Mouse). **A.1,B.1,C.1**. Probability scores are calculated using the LS approach when the distance between TISs is not considered (equivalent to k=1). **A.2,B.2,C.2** Probability scores are calculated using the LS approach with the distance factor k that best redressed the regression slope as described in Supplementary Text S1. Transcripts with two TISs without an in-frame stop codon between the first TIS and second TIS were used. A 3' artificial start value of R_u=0.05 R_{LTM}-R_{CHX} was used for the Lee *et al.* [4] and a 3' artificial start value of R_u=50 #Harr FPs was used for the Ingolia *et al.* [3] data.

A

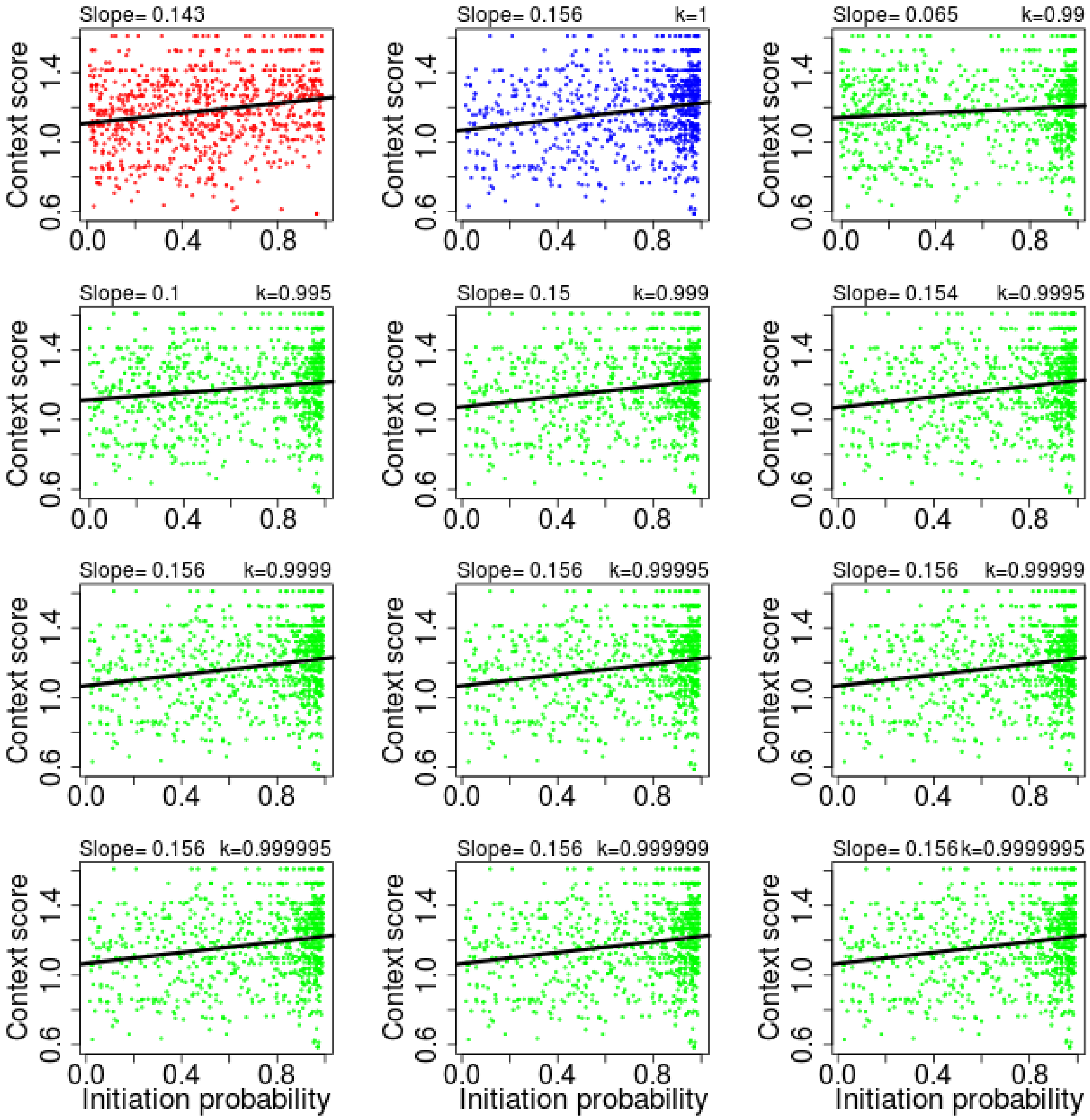

Slope= 0.143
Slope= 0.156
k=1
Slope= 0.065
k=0.99
Slope= 0.1
k=0.995
Slope= 0.15
k=0.999
Slope= 0.154
k=0.9995
Slope= 0.156
k=0.9999
Slope= 0.156
k=0.99995
Slope= 0.156
k=0.99999
Slope= 0.156
k=0.999995
Slope= 0.156
k=0.999999
Slope= 0.156
k=0.9999995
Context score
Initiation probability
0.6
1.0
1.4
0.0
0.4
0.8

B

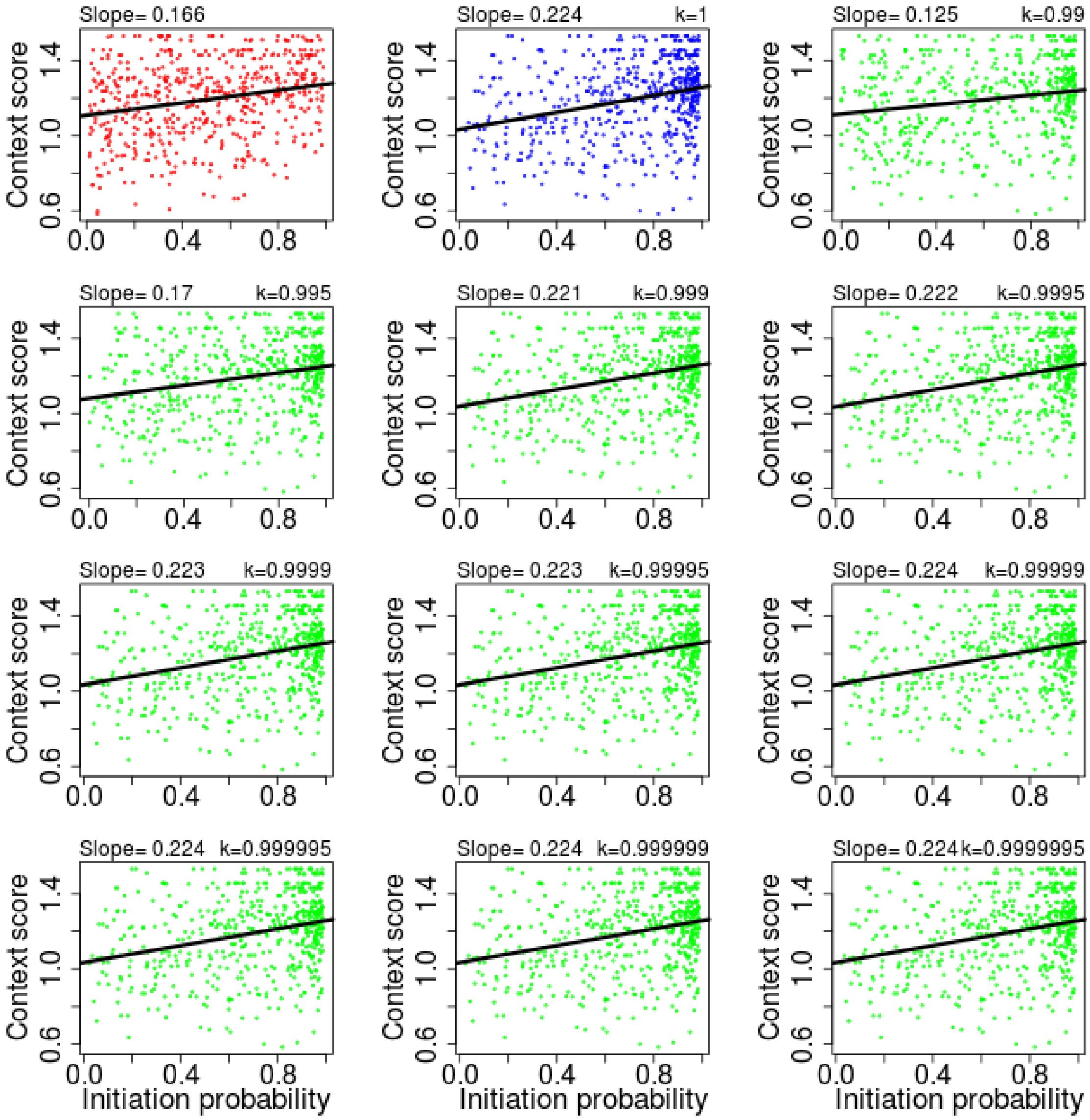

Slope= 0.166
Slope= 0.224
k=1
Slope= 0.125
k=0.99
Slope= 0.17
k=0.995
Slope= 0.221
k=0.999
Slope= 0.222
k=0.9995
Slope= 0.223
k=0.9999
Slope= 0.223
k=0.99995
Slope= 0.224
k=0.99999
Slope= 0.224
k=0.999995
Slope= 0.224
k=0.999999
Slope= 0.224
k=0.9999995
Context score
0.6
1.0
1.4
0.0
0.4
0.8
Initiation probability

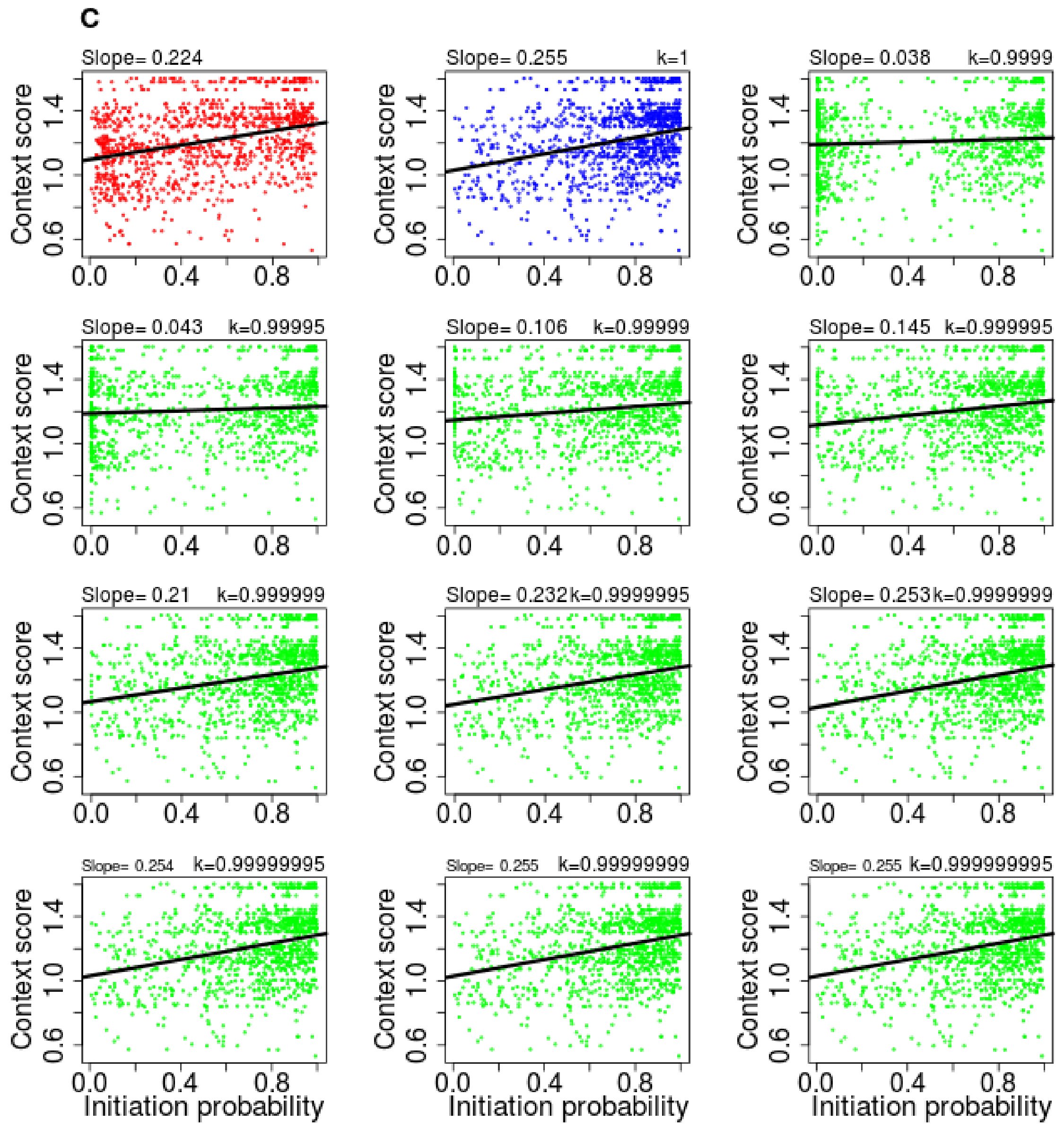

Figure S7. Exploration of the effects of varying the distance parameter k on Kozak context discrimination. **A.** The red plot provides the slope obtained from regressing Kozak context scores onto initiation probability scores estimated using the proportion of footprints for the TISs (PAS method, equation (1) in Results, main text). The blue plot provides the slope for the LS approach (equation (4), main text) when distance is not accounted for (equivalent to k=1). The slopes in the green plots are for different values of the distance parameter k using the LS method (equation (4)). The plots are generated for Human (Lee *et al.* [4] data) and a 3' artificial start value of R_u=0.05 R_{LTM}-R_{CHX} was used. **B.** Generated for Mouse (Lee *et al.* [4] data) with the same description as panel A. **C**. Generated for Mouse (Ingolia *et al.* [3] data) with the same description as panel A except that a 3' artificial start value of R_u=50 #Harr FPs was used.

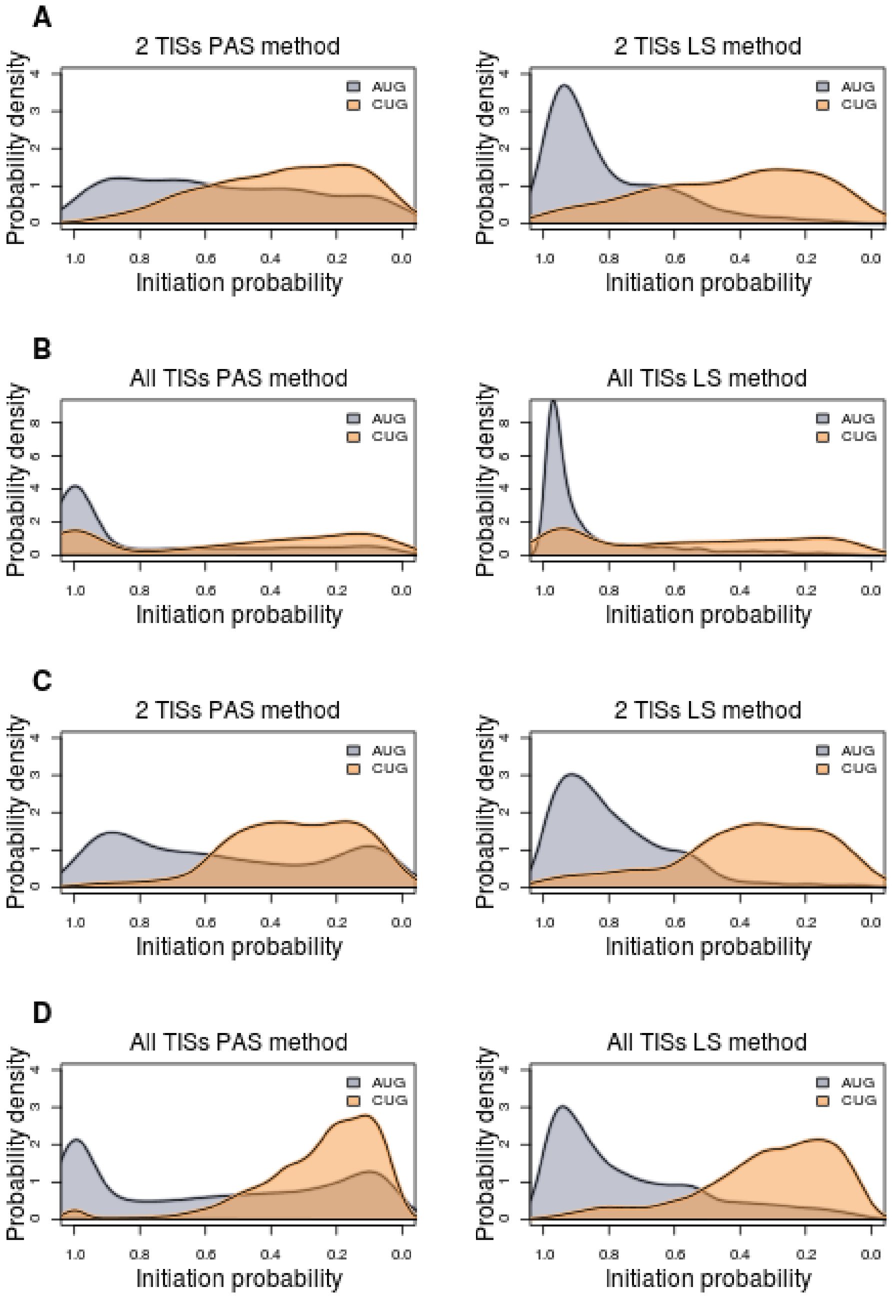

Figure S8. Comparison of methods for discriminating the strength of AUG TISs from non-AUG TISs. **A.** Probability density plots for mouse TISs (Lee *et al.* [4] data) depending on their initiation strength (gray AUG, orange CUG). Left: The scores are calculated as a fraction of the footprints aligning to the TISs from the total number of footprints aligning to the corresponding mRNA (PAS method, equation (1) in Results, main text). Right: The translation initiation probability scores are calculated using the LS method (equation (4) in Results, main text). **B**. Probability density plots for mouse TISs from all mRNAs (left: PAS method, equation (1); right: LS method, equation (4)). **C,D** same as A,B respectively for mouse data from Ingolia *et al.* [3]. For the LS method, a 3' artificial start value of R_u=0.05 R_{LTM}-R_{CHX} was used for the Lee *et al.* [4] and a 3' artificial start value of R_u=50 #Harr FPs was used for the Ingolia *et al.* [3] data.

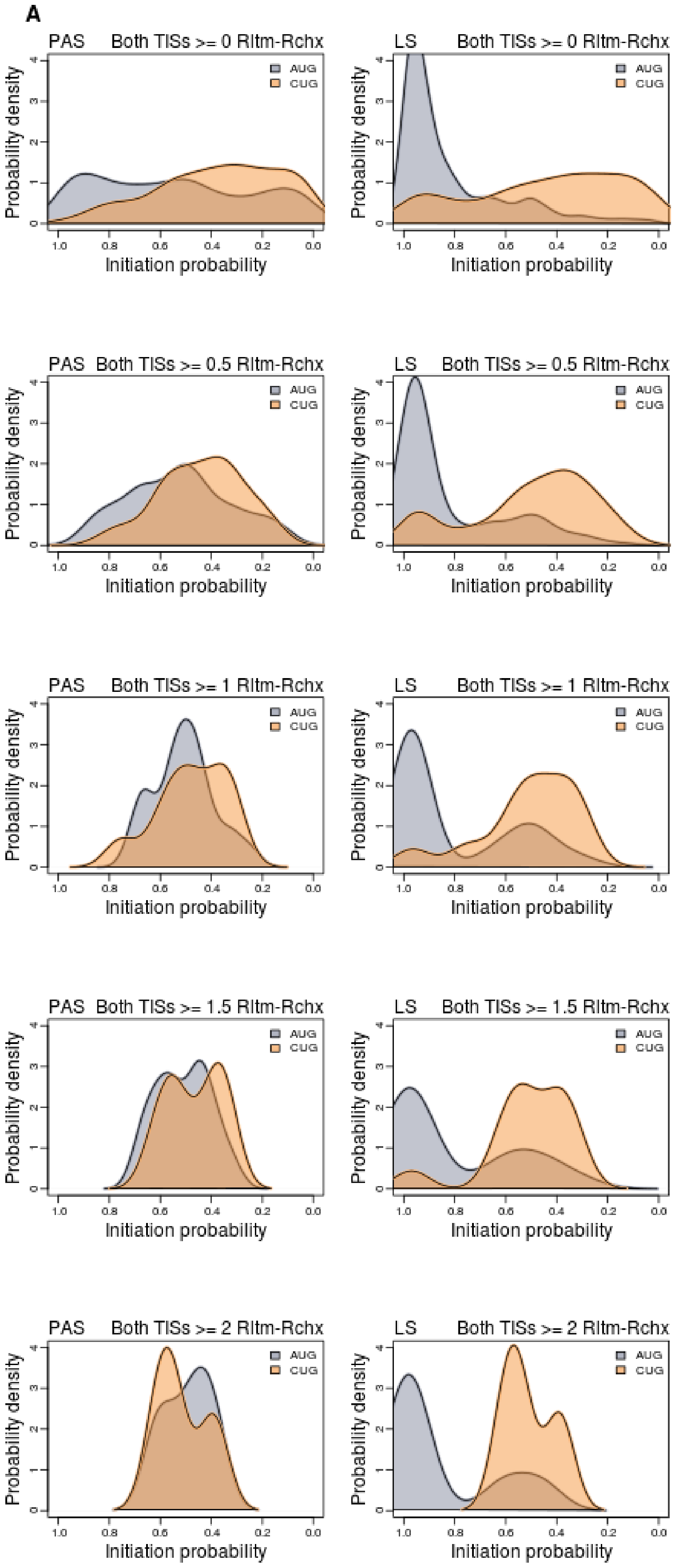
A
PAS Both TISs >= 0 Rltm-Rchx
LS Both TISs >= 0 Rltm-Rchx
PAS Both TISs >= 0.5 Rltm-Rchx
LS Both TISs >= 0.5 Rltm-Rchx
PAS Both TISs >= 1 Rltm-Rchx
LS Both TISs >= 1 Rltm-Rchx
PAS Both TISs >= 1.5 Rltm-Rchx
LS Both TISs >= 1.5 Rltm-Rchx
PAS Both TISs >= 2 Rltm-Rchx
LS Both TISs >= 2 Rltm-Rchx
AUG
CUG
Probability density
Initiation probability

B

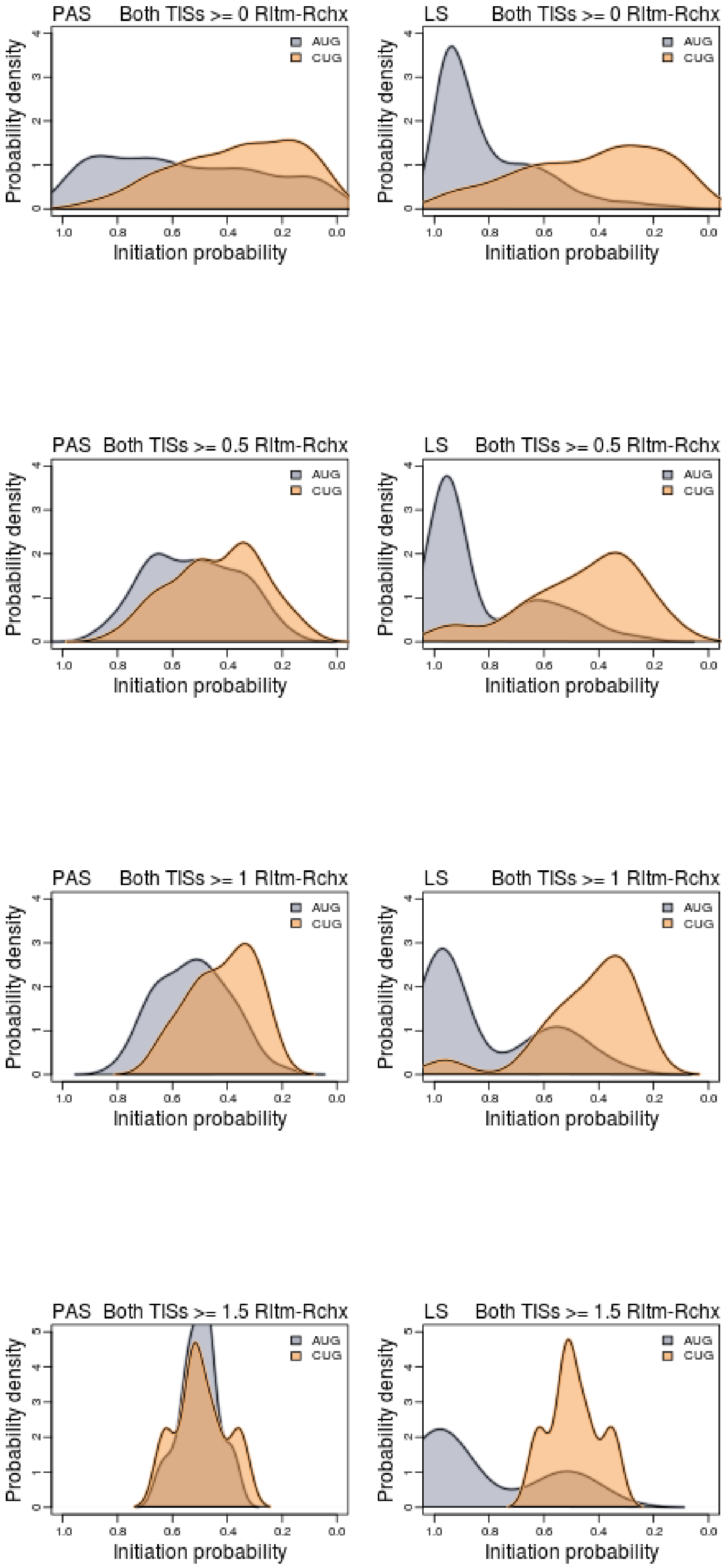
PAS Both TISs >= 0 Rltm-Rchx
LS Both TISs >= 0 Rltm-Rchx
PAS Both TISs >= 0.5 Rltm-Rchx
LS Both TISs >= 0.5 Rltm-Rchx
PAS Both TISs >= 1 Rltm-Rchx
LS Both TISs >= 1 Rltm-Rchx
PAS Both TISs >= 1.5 Rltm-Rchx
LS Both TISs >= 1.5 Rltm-Rchx
AUG
CUG
Probability density
Initiation probability
1.0
0.8
0.6
0.4
0.2
0.0

C

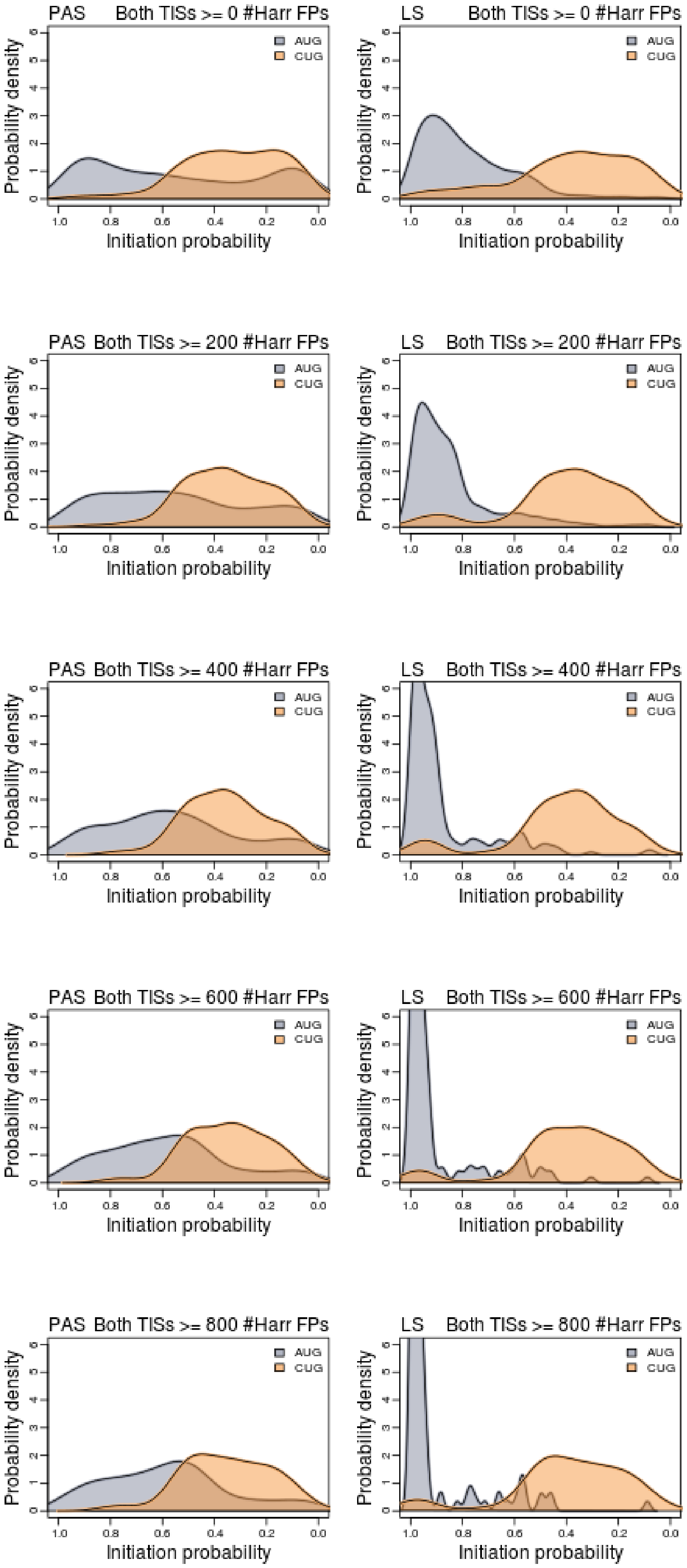
PAS Both TISs >= 0 #Harr FPs
LS Both TISs >= 0 #Harr FPs
PAS Both TISs >= 200 #Harr FPs
LS Both TISs >= 200 #Harr FPs
PAS Both TISs >= 400 #Harr FPs
LS Both TISs >= 400 #Harr FPs
PAS Both TISs >= 600 #Harr FPs
LS Both TISs >= 600 #Harr FPs
PAS Both TISs >= 800 #Harr FPs
LS Both TISs >= 800 #Harr FPs
AUG
CUG
Probability density
Initiation probability
1.0 0.8 0.6 0.4 0.2 0.0

Figure S9 Exploration of how the PAS and LS methods perform under different footprint coverage thresholds. **A.** Probability density plots for human TISs (Lee *et al.* [4] data) depending on their initiation strength (gray AUG, orange CUG) in the 2TISs mRNA dataset where both TISs were greater than or equal to the indicated R_{LTM}-R_{CHX} footprint coverage threshold. Left: The scores are calculated as a fraction of the footprints aligning to the TISs from the total number of footprints aligning to the corresponding mRNA (PAS method, equation (1) in Results, main text). Right: The translation initiation probability scores are calculated using the LS method (equation (4) in Results, main text). A 3' artificial start value of R_u=0.05 R_{LTM}-R_{CHX} was used. **B.** Generated for Mouse (Lee *et al.* [4] data) with the same description as panel A. **C**. Generated for Mouse (Ingolia *et al.* [3] data) with the same description as panel A except for different #Harringtonine footprint coverage thresholds and a 3' artificial start value of R_u=50 #Harr FPs.

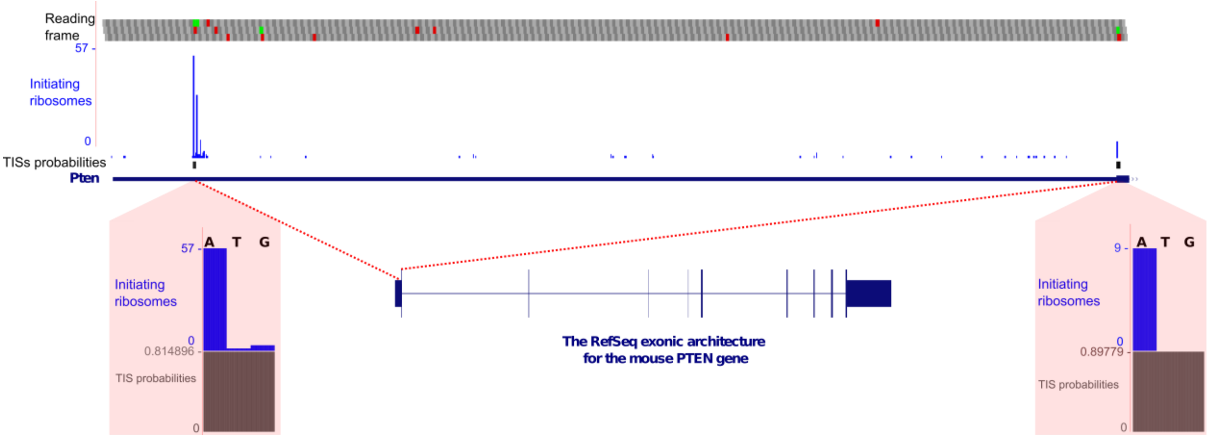

Figure S10 TISs initiation probability browser tracks in GWIPS-viz (http://gwips.ucc.ie/). Visualization of two AUG TISs for the PTEN gene in mouse from GWIPS-viz (generated from Lee *et al.* [4] data). As can be seen from the reading frames, the first AUG TIS originates from an uORF (green bars represent AUGs and the red bars represent stops). The second AUG TIS denotes the annotated start codon for PTEN. An alternative translation initiation site at an upstream CUG codon in-frame with the canonical AUG translation initiation codon [27] was not detected under the conditions of the Lee *et al.* [4] study.
